# Upconversion superballs for programmable photoactivation of therapeutics

**DOI:** 10.1038/s41467-019-12506-w

**Published:** 2019-10-08

**Authors:** Zhen Zhang, Muthu Kumara Gnanasammandhan Jayakumar, Xiang Zheng, Swati Shikha, Yi Zhang, Akshaya Bansal, Dennis J. J. Poon, Pek Lim Chu, Eugenia L. L. Yeo, Melvin L. K. Chua, Soo Khee Chee, Yong Zhang

**Affiliations:** 1 Faculty of Engineering, Department of Biomedical Engineering, National University of Singapore, Singapore, 117583 Singapore; 2 NUS Graduate School for Integrative Sciences and Engineering, National University of Singapore, Singapore, 117456 Singapore; 3 Division of Radiation Oncology, National Cancer Centre Singapore, Singapore, 169610 Singapore; 4 Oncology Academic Program, Duke-NUS Medical School, Singapore, 169857 Singapore; 5 Division of Medical Sciences, National Cancer Centre Singapore, Singapore, 169610 Singapore; 6 Division of Surgical Oncology, National Cancer Centre Singapore, Singapore, 169610 Singapore

**Keywords:** Cancer therapy, RNAi, Bioinspired materials, Nanoparticles

## Abstract

Upconversion nanoparticles (UCNPs) are the preferred choice for deep-tissue photoactivation, owing to their unique capability of converting deep tissue-penetrating near-infrared light to UV/visible light for photoactivation. Programmed photoactivation of multiple molecules is critical for controlling many biological processes. However, syntheses of such UCNPs require epitaxial growth of multiple shells on the core nanocrystals and are highly complex/time-consuming. To overcome this bottleneck, we have modularly assembled two distinct UCNPs which can individually be excited by 980/808 nm light, but not both. These orthogonal photoactivable UCNPs superballs are used for programmed photoactivation of multiple therapeutic processes for enhanced efficacy. These include sequential activation of endosomal escape through photochemical-internalization for enhanced cellular uptake, followed by photocontrolled gene knockdown of superoxide dismutase-1 to increase sensitivity to reactive oxygen species and finally, photodynamic therapy under these favorable conditions. Such programmed activation translated to significantly higher therapeutic efficacy in vitro and in vivo in comparison to conventional, non-programmed activation.

## Abstract (web summary)

The synthesis of nanoparticles capable of orthogonal photo-activity is complex. Here, the authors report on an assembly process of two distinct nanoparticles into composite nanoparticles as a simple method and report on the application in photodynamic therapy and siRNA delivery in tumour therapy.

## **I**ntroduction

Understanding the basic unit of life has remained a formidable challenge for centuries. Though we have come a long way in understanding the physico-chemical properties of the building blocks, such as DNA, RNA, lipids, and proteins, our understanding of their highly complex intramolecular interactions, which helps run the cellular machinery is still primitive. So, there is an immense need to develop modern tools, which can analyze the spatio-temporal dynamics of these complex interactions to understand their function and subsequently to control them for therapeutic interventions. Light-based tools and techniques are highly preferred to regulate/impede these biological processes and investigate/control their response due to their spatio-temporal specificity, precision of activation and tunable dynamics. Various light-based techniques, such as light-controlled ion channels^1^, photoactivated protein expression/targeting/cleavage^2^, photoactivated RNAi^3^, photoactivated release of biomolecules^4^, and photodynamic therapy^5^, have been used for elucidation of cellular functions and for therapeutic applications. However, majority of light-sensitive biomolecules/chemicals are only sensitive to UV and visible light, which suffer from limitations, such as phototoxicity (UV) and low tissue penetration capabilities (UV/visible)^5^, thereby restricting their use to an in vitro setting. Near infrared (NIR) light-excitable upconversion nanoparticles (UCNPs) are one of the most preferred nanoplatforms for in vivo applications, as they can act as a nanotransducer to absorb NIR light with low phototoxicity and high tissue penetration depth and convert it to UV/visible light^6–8^. Such upconverted light has then be shown to be used successfully in various applications ranging from optogenetics^9–11^, photodynamic therapy (PDT)^6,12,13^, photoactivated drug/biomolecule release^14–16^ and photo-controlled gene expression^8,17,18^ in vitro and in vivo. Traditional UCNPs with multiple emissions under excitation at a single wavelength (980 or 808 nm) can be used for simultaneous activation of single or multiple biomolecules/drugs only, but this is not programmable. So far UCNPs have not been used for programmable control of biological processes. However, various biological processes need temporally separated and precise photoactivation of different molecules. So, development of UCNPs with emissions at different wavelengths when excited at 980 and 808 nm, respectively, for programmable and orthogonal photoactivation of multiple molecules is of utmost necessity and interest.

Efforts have been taken to develop orthogonal UCNPs, which could be excited under different wavelengths to produce different emissions. This was achieved by producing core–shell-structured UCNPs with multiple shells and isolating different lanthanide activator/emitter ions in different shells of the nanocrystal to prevent cross-relaxation quenching. However, epitaxial growth of different shell layers is a challenging and time-consuming process, and successful coating of the shells reduces exponentially with the increase in the number of shells. To overcome this problem, we have devised a strategy to modularly assemble UCNPs to achieve orthogonal emissions, which allows for the synthesis of individual modules (UCNPs) separately and assemble them together to form orthogonal photoactivable-superballs (OP-SBs). The individual UCNPs are designed to have strong red or UV/blue emissions upon excitation at 980 and 808 nm, but not both. This allows for different lanthanide activator/emitter ions to be placed in different regions of the OP-SBs instead of in different epitaxially grown shells. It offers great flexibility in tweaking parameters such as the number of individual UCNPs in each SB, their size/shape/fluorescence emissions/intensity of emissions etc.

These developed OP-SBs were then used to demonstrate programmable/orthogonal photoactivation by enhancing the efficiency of PDT. UCNPs have been used for NIR-based PDT with relative success but their effectiveness as a standalone treatment is low. Primary reasons for the lower efficiency of PDT could be due to the recycling of the nanoparticles by the cellular machinery^18,19^ and the inherent ability of the cells to withstand and combat free radicals^19,20^. These problems were targeted by employing photochemical internalization to enable endosomal escape and prevent them from being recycled by the cells and the inherent resistance against free radicals was targeted by knocking down the gene for superoxide dismutase 1 (SOD1), which is responsible for destroying free radicals^21,22^. After the OP-SBs have escaped from the endosomes and caused knockdown of SOD1, the cells would be subjected to PDT. However, it is very important that these processes have to be activated in a sequential manner, with the right treatment at the right time for the right duration and this requires programmable and orthogonal photoactivation capabilities, which is not available in conventional UCNPs. If the siRNA is released during photochemical internalization (PCI), the siRNA might lose part of its functionality due to the low pH conditions in the endosome and if the PDT is performed along with siRNA release, there is no time available for gene knockdown and the cells might clear the free radicals produced during PDT. Hence, the nanoparticles should first escape the endosomes via PCI for the gene knockdown to be effective and optimal gene knockdown should be achieved before PDT can be performed to obtain better cell killing efficiency.

The as-prepared OP-SBs were loaded with a photosensitizer (Zinc phthalocyanine, ZnPc) for PCI/PDT and SOD1 siRNA for gene knockdown. The release of siRNA from the OP-SBs is controlled by azobenzene-based light activated caps on the mesopores of the OP-SBs under 808 nm irradiation. The ZnPc is activated by the OP-SBs under 980 nm irradiation. Short durations of 980 nm irradiation results in PCI and longer durations of 980 nm irradiation results in PDT. Initially the ability of the OP-SBs to activate ZnPc and siRNA release individually and in a programmed fashion is tested in solution. This is followed by programmed activation of these therapeutics in vitro in 2D and 3D cultures (cervical cancer and oral cell carcinoma model) and in vivo (human oral cell carcinoma model). The OP-SBs are also biocompatible and did not show any significant toxicity in vitro and in vivo.

## Results

### Design and synthesis of OP-SBs

**Fig. 1:**
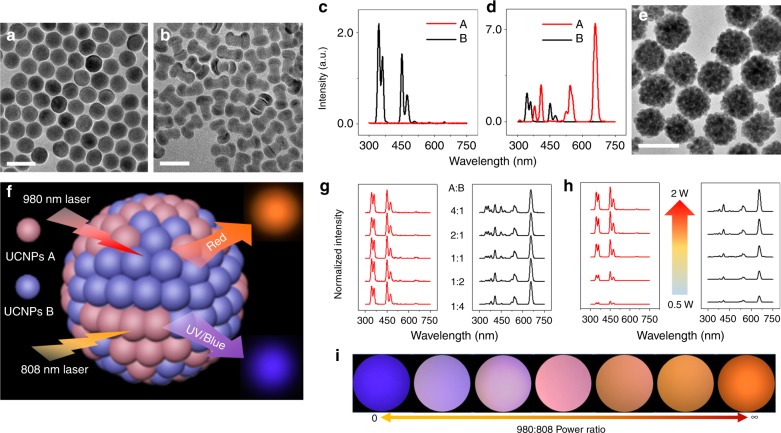
Orthogonal excitation superballs with mixed UCNPs A and B. TEM images of UCNPs A (**a**) and UCNPs B (**b**). 808 nm (**c**) laser and 980 nm laser (**d**) excited upconversion emission spectra of cyclohexane solutions at room temperature (25 °C) comprising A (red line) and B (black line) with same concentration. **e** TEM images of OP-SBs. **f** Schematic illustration of OP-SBs with luminescent photos taken by smart phone camera. **g** Normalized 808 nm laser (left) and 980 nm laser (right) excited upconversion emission spectra of superball aqueous suspension at room temperature (25 °C) with different mixing ratio between A and B. 808 and 980 nm laser power (1 W) are kept same throughout the measurement. **h** 808 nm laser (left) and 980 nm laser (right) excited upconversion emission spectra with increasing laser power of superball (A:B = 1:1) aqueous suspension at room temperature (25 °C). **i** Smartphone images of OP-SBs (A:B = 1:1) pattern under different 980 nm:808 nm laser power ratio. Scale bar: 50 nm for **a**, **b**, 200 nm for **e**. UCNPs A, NaYF_4_: 60%Yb, 20%Gd, 2%Er@NaLuF_4_: 25%Y; UCNPs B, NaYF_4_: 30Yb, 0.5%Tm@NaYF_4_: 10%Yb@NaNdF_4_: 10%Yb

Taking advantage of our modular concept, we designed OP-SBs, which had red emission under 980 nm excitation and blue/UV emission under 808 nm laser excitation to coincide with the excitation of the photosensitizer and the light-activated caps. We designed a core–shell UCNPs (A) with intense red emission under 980 nm. UCNPs A had relatively high Yb^3+^ doping concentration (60%) compared with commonly used NaYF_4_: Yb, Er UCNPs (20%). The high doping concentration of Yb^3+^ brings two virtues to the system: high Yb^3+^ doping will enable more cross-relaxation of energy between the 4f energy states, redistribute and concentrate energy to the ^4^f_9/2_ states of Er^3+^, resulting in intense red emission; on the other hand, high Yb^3+^ doping concentration will render the UCNPs with very strong 980 nm photon absorption ability and upconversion luminescence under 980 nm laser and hence will cover the emission from other counterparts. Next, a commonly used core-shell-shell UCNPs (B) base on Nd^3+^ sensitization, that produces UV/blue emissions under 808 nm excitation was synthesized. The composition and structural details of the two UCNPs are specified in Supplementary Table 1. The UCNPs A and B used here were of spherical and dumbbell shapes respectively, with uniform size distribution, as observed from their TEM (Fig. 1a, b). Spectrums of UCNPs A and B in cyclohexane suspension showed that, under 808 nm laser excitation, B showed strong blue and UV emission while no signal was observed from A. On the other hand, under 980 nm laser excitation, A showed red emission, which was much stronger than the blue and UV emissions from B (Fig. 1c, d).

An emulsion-based synthesis method was used to design the modular OP-SBs as illustrated in Supplementary Fig. 1 by mixing UCNPs A and B in different ratios. A stable oil-in-water emulsion system was obtained under vigorous stirring and sonication, by which the oleic acid-capped UCNPs A and B were confined in the emulsion droplets that were stabilized by the surfactants present in the aqueous phase. Subsequently, the low-boiling cyclohexane was evaporated from the oil emulsion droplets by heating the solution at 70 °C. During solvent removal, the droplets shrink and the UCNPs in the droplets get concentrated and pack closely with each other through hydrophobic Van der Waals interactions of oleic acid, thereby assembling to form OP-SBs. Formation of OP-SBs was evaluated using TEM and dynamic light scattering (DLS). The hydrodynamic diameter of the OP-SBs was found to be around 170 nm (Supplementary Fig. 1c, solid line), which was significantly (four times) larger than the individual UCNPs’ diameter of around 40 nm (Supplementary Fig. 1c, dash line). From the TEM images in Fig. 1e, it can be seen that the UCNPs have assembled together to form spherical OP-SBs. From the magnified images (Supplementary Fig. 1d), it can be observed that the UCNPs did not sinter into larger units and their individual morphology was retained. As expected, two different UCNPs were found to be preserved within the superball, implying that the superball is assembled from a mixture of A and B (Supplementary Fig. 1d). The orthogonal photoactivation behavior (Fig. 1f) of the OP-SBs were then investigated. OP-SBs with five different mixing ratios of A:B were prepared to study the relationship between precursor UCNPs’ ratio and their luminescence. DLS data revealed that the sizes of all the five samples were similar to each other (Supplementary Fig. 2), ensuring the success of the assembly. After this, luminescence emission spectra were recorded under 980 and 808 nm laser excitation (Fig. 1g) and the OP-SBs were found to have a well-maintained orthogonal luminescence in all the five ratios tested. It is worth to note that, as a filtration effect from the high Yb^3+^ doping concentration in A, upconversion luminescence from B under 980 nm excitation was further reduced, rendering a more perfect orthogonal emission behavior to the OP-SBs^23^.

Henceforth, studies were performed on OP-SBs with A:B = 1:1. The emission spectrum of OP-SBs suspension was further measured with different laser powers to assess their impact on the orthogonal luminescence and its tunability. Results showed that the orthogonal luminescence was maintained for all laser powers tested and the emission intensity was closely related to laser power (Fig. 1h). To have an indicative idea that the final color of OP-SBs is determined by both 808 and 980 nm laser, pictures were taken with 808 nm:980 nm laser power ratio varying from 0 (completely 808 nm laser excitation, Fig. 1i left) to infinite (completely 980 nm laser excitation, Fig. 1i right). As the laser power ratio changed, color of the pattern gradually shifted from blue to orange when observed using a smartphone camera (Supplementary Fig. 3). Besides the UCNPs pairs (A and B) used above, other OP-SBs were also prepared using some other UCNPs pairs and the orthogonal luminescence were tested and recorded to show that this effect is not restricted to a certain type of UCNPs (Supplementary Fig. 4).

### Programmed orthogonal photoactivation using OP-SBs

The OP-SBs were firstly coated with a thin layer of mesoporous silica for loading of cargo molecules as shown in Supplementary Fig. 5. The mesoporous silica-coated OP-SBs were then modified with photosensitive azobenzene-based caps, which swivels and releases the cargo inside the mesopores upon UV/blue light irradiation^24^. Azobenzene-modified OP-SBs (OP-SBs@azo) were characterized using UV-Vis spectrophotometry to ensure the modification of photosensitive caps on the OP-SBs (Supplementary Fig. 6). The OP-SBs@azo were then loaded with ZnPC and siRNA (OP-SBs@azo-Psi). The hydrodynamic diameter, zeta potential and loading content of ZnPc and siRNA is given in Supplementary Table 2. The loading efficiency of ZnPC and siRNA on the OP-SBs@azo-Psi was measured by absorbance and fluorescence spectrophotometry, respectively, and found to be 50.66% and 63.21%.

**Fig. 2:**
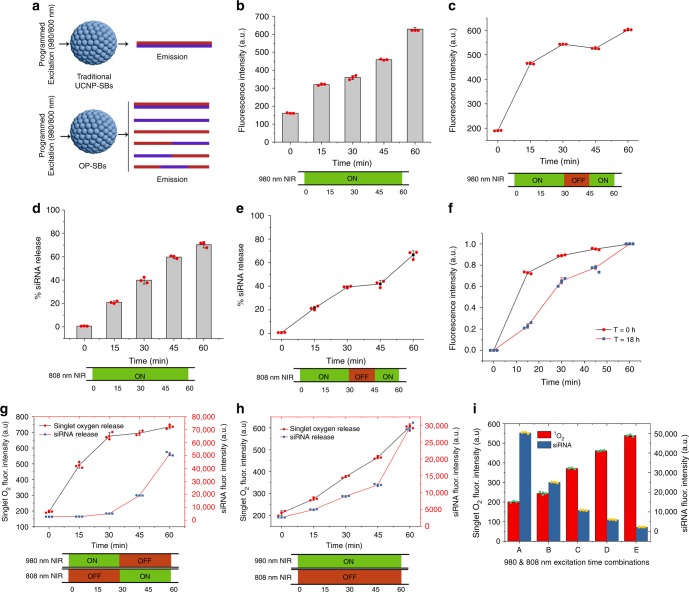
Orthogonal photoactivation of multiple therapeutics. **a** Schematic showing the difference in emission profile between Control-SBs@azo-Psi and OP-SBs@azo-Psi with programmed activation using 980/808 nm laser. **b** Release of singlet oxygen from OP-SBs@azo-Psi in solution over time with 980 nm irradiation. **c** Step-wise release of singlet oxygen from OP-SBs@azo-Psi in solution with 980 nm irradiation. **d** Release of siRNA from OP-SBs@azo-Psi in solution over time with 808 nm irradiation. **e** Step-wise release of siRNA from OP-SBs@azo-Psi in solution with 808 nm irradiation. **f** Singlet oxygen production using 980 nm excitation of the same OP-SBs@azo-Psi at different time points. **g** Orthogonal activation of singlet oxygen production and siRNA release from OP-SBs@azo-Psi over time. **h** Non-orthogonal activation of singlet oxygen production and siRNA release from Control-SBs@azo-Psi over time. **i** Programmed activation of OP-SBs@azo-Psi with different durations of 980 and 808 nm NIR irradiation. Error bars represent the standard deviation of measurements from three (*n* = 3) distinct samples

First, the production of singlet oxygen (^1^O_2_) and siRNA release from OP-SBs@azo-Psi was performed in solution in a controlled manner. Figure 2a illustrates the innumerable types of programmed emissions which could be obtained by the activation of OP-SBs@azo-Psi, whereas only a single emission profile could be obtained from traditional Control-SBs@azo-Psi even with programmed activation. OP-SBs@azo-Psi were mixed with a ^1^O_2_ sensitive dye (^1^O_2_ sensor green) and the production of ^1^O_2_ in solution was studied when they were irradiated with a 980 nm NIR laser continuously and discontinuously. It can be seen from Fig. 2b that the OP-SBs@azo-Psi had a steady production of ^1^O_2_ over time and there is a step-wise increase in the production of ^1^O_2_ if the irradiation is discontinuous (Fig. 2c). A similar trend was observed for the siRNA release when the OP-SBs@azo-Psi was irradiated with an 808 nm NIR laser (Fig. 2d, e). These results demonstrate the ability of the OP-SBs@azo-Psi to release cargo on-demand separately. Next, the ability of the OP-SBs@azo-Psi to produce ^1^O_2_ repeatedly at different time points was tested. This is required, as the same nanoparticle should be able to produce ^1^O_2_ initially for endosomal escape and then after 18 h to enable cell killing. So, the same solution of OP-SBs@azo-Psi was irradiated at different time points (*t* = 0 h and *t* = 18 h) by a 980 nm NIR laser and the production of ^1^O_2_ was tested. As seen from Fig. 2f, the OP-SBs@azo-Psi were able to produce ^1^O_2_ efficiently in a repeated manner. Furthermore, Supplementary Fig. 7 ascertained that the ^1^O_2_ production under 808 nm NIR excitation is not significant in comparison to the 980 nm NIR excitation, hence assuring orthogonal control over the different processes. Next the OP-SBs@azo-Psi were irradiated with 980 nm NIR laser for 30 mins and 808 nm NIR laser for 30 mins. It was seen that the ^1^O_2_ production and siRNA release could be activated independently as shown in Fig. 2g. This has not been possible before with the use of traditional UCNPs which can activate multiple processes simultaneously under either 980 or 808 nm excitation, but not subsequently in a programmable/orthogonal fashion. This is evident from Fig. 2h where Control-SBs@azo-Psi (excitable by 980 nm laser and having emission wavelengths corresponding to siRNA release and ^1^O_2_ production) were used and the release of ^1^O_2_ and siRNA from them was studied. In this case, orthogonal activation was not possible and the release of siRNA and ^1^O_2_ occured in a simultaneous fashion. Finally, the OP-SBs@azo-Psi was activated with different ratios of 980 and 808 nm NIR lasers amounting to 60 mins and the effect was studied. As observed from the results in Fig. 2i, it can be seen that the production of ^1^O_2_ and siRNA release, resulting from the excitation of two different lasers can be controlled specifically to cater for particular applications.

### Photoactivation of therapeutic processes in vitro

**Fig. 3:**
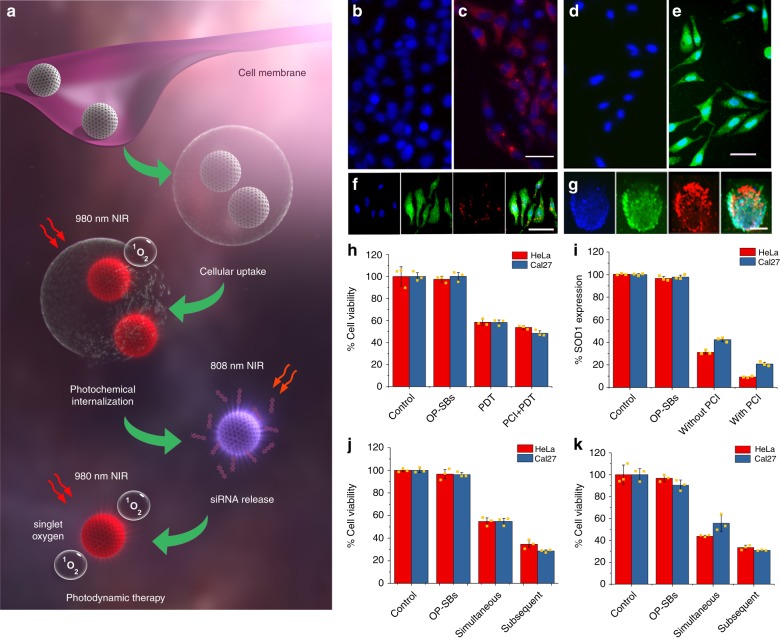
Programmed orthogonal photoactivation. **a** Schematic illustration of orthogonal excitation of photosensitizers and azobenzene-based caps for endosomal escape, siRNA release and photodynamic therapy. **b**, **c** Cells without and with siRNA loaded OP-SBs and irradiated with 808 nm NIR for siRNA release, scale bar is 50 μm. **d** Production of singlet oxygen in cells with NIR irradiation in comparison to non-irradiated cells (**e**), scale bar is 50 μm. **f**, **g** Uptake of OP-SBs@azo-Psi in 2D culture cells (scale bar is 50 μm) and 3D tumor spheroids (scale bar is 200 μm). **h** Comparison of HeLa and Cal27 cell killing by OP-SBs@azo-Psi based PDT without and with PCI. **i** Comparison of SOD1 gene expression without and with PCI. **j**, **k** Comparison of simultaneous (non-orthogonal) and subsequent (orthogonal) activation of endosomal escape, siRNA release and PDT and their effects on cell viability of HeLa and Cal27 2D culture and 3D tumor spheroids. Error bars represent the standard deviation of measurements from three (*n* = 3) distinct samples

The schematic showing the process of orthogonal photoactivation by OP-SBs@azo-Psi to achieve enhanced PDT is shown in Fig. 3a. Once the OP-SBs@azo-Psi are internalized by the cells via endocytosis, they are made to escape the endosome by PCI. This is done by irradiating the OP-SBs@azo-Psi with 980 nm NIR laser. The ^1^O_2_ produced by the nanoparticle disrupts the endosomes and releases the nanoparticles into the cytosol and prevents their recycling. Once the OP-SBs@azo-Psi reaches the cytosol, the siRNA loaded onto the nanoparticles are released by irradiation with an 808 nm NIR laser, which in turn activates the azobenzene caps on the nanoparticles thereby triggering the siRNA release. Finally, after the gene knockdown has started taking effect, the OP-SBs@azo-Psi were irradiated with a 980 nm NIR laser to produce ^1^O_2_ for PDT. The following time points were chosen for photoactivation of the various processes; PCI was activated 8 h post incubation (when OP-SBs@azo-Psi uptake was high, Supplementary Fig. 8), siRNA release was activated at 8.5 h (endosomal escape is instantaneous but a 30 mins gap was given for the OP-SBs@azo-Psi to distribute well in the cytosol) and PDT was performed at 26 h (minimum of 16 h for SOD1 knockdown to take effect).

Initially, the OP-SBs@azo-Psi were tested for their serum stability and cytotoxicity. It was seen that the OP-SBs@azo-Psi were highly stable in water and in 10% FBS over a period of 72 h as shown in Supplementary Fig. 9. They exhibited negligible toxicity in concentration ranges up to 500 µg/mL and minimal toxicity in higher concentrations tested as observed from Supplementary Fig. 10 and 500 µg/mL was chosen for further in vitro experiments. The NIR light used in this study also conferred negligible/minimal phototoxicity to cells as shown in Supplementary Fig. 11. The cell viability rather increased with 808 nm NIR irradiation minimally due to photobiostimulation effects. After initial characterization studies, the mechanism of OP-SBs@azo-Psi entry into cells was studied by incubating OP-SBs@azo-Psi with cells treated with various inhibitors of cellular uptake including Cytochalasin B, nystatin, filipin and chlorpromazine corresponding to micropinocytosis, lipid-raft-mediated uptake, caveolae mediated uptake and clathrin-mediated uptake respectively. As seen from Supplementary Fig. 12, the OP-SBs@azo-Psi predominantly entered the cell through clathrin-mediated endocytosis as there was a significant drop in cellular uptake with chlorpromazine treatment (*p* < 0.01, *p* = 0.00102) and minimally through micropinocytosis.

The individual components of the therapeutic process involving multiple photoactivations were tested initially. The siRNA release from the nanoparticles was assessed by using fluorescent tagged siRNA and utilizing an 808 nm NIR laser to trigger the release of siRNA from the OP-SBs@azo-Psi. The release was then monitored by fluorescence microscopy and quantified by flow cytometry. The un-treated cells do not have any siRNA fluorescence (Fig. 3b) and the treated cells without NIR irradiation shows minimal fluorescence (Supplementary Fig. 13) which could be due to the baseline release of the siRNA without any light trigger. However, the OP-SBs@azo-Psi treated cells with 808 nm NIR irradiation shows enhanced and well-distributed siRNA luminescence in the cytosol (Fig. 3c) confirming the release of siRNA from the OP-SBs@azo-Psi. A similar trend was observed when the siRNA release was quantified using flow cytometry as shown in Supplementary Fig. 14. There was a 60-fold increase in the fluorescence of siRNA in cells with 808 nm NIR treatment in comparison to cells without NIR treatment. Then, the production of ^1^O_2_ by the OP-SBs@azo-Psi for PDT was assessed by staining the cells with a ^1^O_2_ sensitive dye (Image-IT™ LIVE Green ROS) and irradiating the cells with a 980 nm NIR laser. From Fig. 3e, it can be seen that there was significant ^1^O_2_ production as observed from the green luminescence in comparison to untreated control cells as shown in Fig. 3d. Subsequently, the uptake of the OP-SBs@azo-Psi was visualized by confocal microscopy in 2D culture (Fig. 3f) as well as in 3D spheres (Fig. 3g) and it was seen that they were effectively taken up by the cells/spheres and uniformly distributed, 8 h post incubation.

Finally, the photoactivation of multiple therapeutics was quantified in two cancer models namely, cervical (HeLa) and head and neck cancer (Cal27) models. First, it was tested if PCI enhances the effect of PDT and it was seen that there was ~8.7% (*p* < 0.05, *p* = 0.0282) and ~17% (*p* < 0.05, *p* = 0.0109) decrease in cell viability of HeLa and Cal27 cells, respectively, when treated with PCI + PDT in comparison to PDT alone (Fig. 3h). Secondly, the effect of gene knockdown with and without PCI was studied. It was observed that there was ~65% (*p* < 0.01, *p* = 0.0051) and ~51% (*p* < 0.01, *p* = 0.000089) decrease in gene knockdown with PCI, in comparison to cells without PCI for HeLa and Cal27 cells, respectively (Fig. 3i). This could be attributed to the siRNA being released into the cytosol rather than in the harsh environment of the endosomes. Thirdly, the OP-SBs@azo-Psi were incubated in cells (2D culture and 3D spheres) and they were orthogonally activated to enable photochemical internalization (980 nm activation), siRNA release (808 nm activation) and PDT (980 nm activation) at 8, 8.5, and 26 h respectively (Fig. 3j, k). This was compared with Control-SBs@azo-Psi which was subjected to photochemical internalization (980 nm activation), siRNA release (980 nm activation) and PDT (980 nm activation) at 8, 8.5, and 26 h respectively. The subsequent (orthogonal) activation resulted in better cell killing, approximately 37% (*p* < 0.05, *p* = 0.0428) and 48% (*p* < 0.01, *p* = 0.000109) higher than that of simultaneous (non-orthogonal) activation by Control-SBs@azo-Psi for HeLa and Cal27 2D cultures respectively. Similarly, for 3D spheres of HeLa and Cal27, the cell killing of subsequent activation was found to be ~23.5% (*p* < 0.01, *p* = 0.00166) and ~44.5% (*p* < 0.01, *p* = 0.00461) higher than simultaneous activation for HeLa and Cal27 spheroids respectively. This could be due to the inability of the Control-SBs@azo-Psi to activate siRNA release and ^1^O_2_ independently. The cell killing starts with the production of ^1^O_2_ even while the siRNA is being released, leaving insufficient time for gene knockdown to take effect. Also, the release of siRNA into the low pH environment of the endosomes during PCI will reduce the functionality of the siRNA, resulting in lower gene knockdown.

### Biodistribution of OP-SBs post-administration in vivo

We then determined the biodistribution of the OP-SBs (OP-SBs@mSiO_2_) in mice by quantifying the concentration and percentage injected dose of Y (Yttrium) and Yb (Ytterbium) in various major organs: liver, spleen, kidneys, lungs, heart, urine, blood and faeces, at 6 h, 24 h, 1 week, and 1 month post injection using inductively coupled plasma mass spectrometry (ICP-MS). At both injection doses of 25 and 50 mg/kg, OP-SBs were cleared from the blood circulation in <6 h post injection and could be detected primarily with a high percentage in the liver and spleen, with a much lower degree of accumulation detected in the lung and kidney. Trace amounts were also detected in the heart, urine and faeces. The biodistribution of Y is given in Supplementary Fig. 15a, b for 25 and 50 mg/Kg of OP-SBs injected respectively. The biodistribution of Yb is given in Supplementary Fig. 15c, d for 25 and 50 mg/Kg of OP-SBs injected, respectively. At the injection dose of 25 mg/kg OP-SBs, consistent accumulation of Yb and Y in the liver and spleen was observed from 6 h to 1 month post injection. However, for mice dosed with 50 mg/kg OP-SBs, a decrease in accumulation of Yb and Y in the liver after 6 h was observed (Yb, *p* = 0.006; Y, *p* = 0.008), particularly when comparing between 6 h and 1 month post injection, indicating the potential clearance of the OP-SBs from the liver. This decrease in nanoparticle concentration following peak accumulation was similarly reported by He et al., who studied the biodistribution of mesoporous silica nanoparticles over time^25^.

With the spleen and liver being major organs of the mononuclear phagocytes system (MPS), the OP-SBs accumulation observed in these organs was likely a result of clearance by spleen macrophages and Kupffer cells in the liver. Following intravenous injection of OP-SBs, these nanoparticles were opsonized and cleared from the blood circulation over time by these macrophages via phagocytosis^26,27^. Size of the OP-SBs also played a role in the rate of accumulation in these MPS organs^25,27^. Collectively, these factors contributed to the characteristics of the OP-SBs biodistribution observed.

### Biocompatibility of OP-SBs in vivo

Following intravenous delivery of OP-SBs in vivo, the body weight gain of the mice observed throughout the treatment period was observed to differ slightly between mice injected with 25 or 50 mg/kg OP-SBs compared to untreated mice (control). A significant weight difference of up to 1.76 g for 25 mg/kg dose (10.5% of mean initial weight) and 1.96 g for 50 mg/kg dose (11.7% of mean initial weight) was observed between day 6 to day 10 (25 mg/kg, day 6, *p* = 0.04822; 25 mg/kg, day 8, *p* = 0.02125; 25 mg/kg, day 10, *p* = 0.0089; 50 mg/kg, day 6, *p* = 0.03415; 50 mg/kg, day 8, *p* = 0.02185; 50 mg/kg, day 10, *p* = 0.01065). Nonetheless, both treated and untreated mice reached the same relative weight by 1 month post injection (Supplementary Fig. 16). This suggested that while the initial introduction of the OP-SBs may have slightly hindered the feeding behaviour of the mice, the effect was temporary without any long-term adverse effects, which support the biocompatibility of the OP-SBs.

**Fig. 4:**
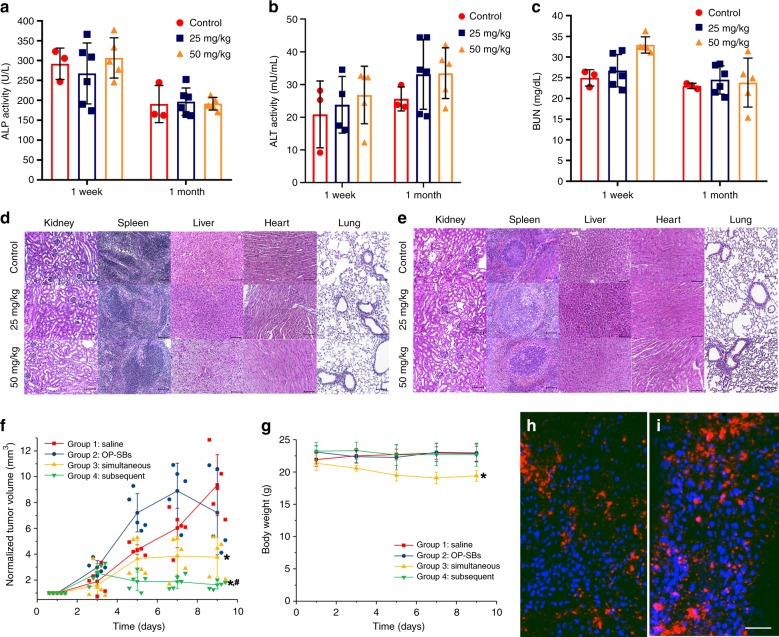
Photoactivation of therapeutic processes using OP-SBs in vivo. Serum levels of alkaline phosphatase (ALP) activity (**a**), alanine aminotransferase (ALT) activity (**b**) and blood urea nitrogen (BUN) (**c**), in mice injected intravenously with OP-SBs at doses of 0 (untreated controls), 25 and 50 mg/kg and sacrificed at 1 week and 1 month post injection. Error bars represent the standard deviation of measurements from three (*n* = 3) distinct samples. **d**, **e** Hematoxylin and eosin staining of tissue sections obtained from the mice major organs at 1 week and 1 month post injection of the OP-SBs, showing that the histological features between mice intravenously injected with OP-SBs of dose 0 (control), 25 and 50 mg/kg, scale bar is 100 μm. **f** Tumor regression studies of Cal27 tumors on Balb/c nude mice injected with different nanoformulations and irradiated with a 980 and/or 808 nm NIR laser. **g** Body weight of treated mice over the treatment period. **h**, **i** Distribution of OP-SBs@azo-Psi in tumor at 8 and 24 h after intratumoral injection, scale bar is 100 μm. Error bars represent the standard deviation of measurements from five (*n* = 5) distinct samples. *p < 0.05 in comparison to Group 1 (ANOVA), *^#^*p* < 0.05 in comparison to Group 1 and 3 (ANOVA)

We quantified the blood platelet factor 4 (PF4) concentration, alkaline phosphatase (ALP), alanine aminotransferase (ALT) activity and urea nitrogen (BUN) level in mouse serum from both OP-SBs-treated and control groups as shown in Fig. 4a–c. The rate of the glomerular filtration directly affects the removal of blood urea^28,29^, while liver damage results in elevation of ALP and ALT metabolic enzymes in the blood^30,31^. Platelet activation and degranulation results in the release of the chemokine PF4 from platelets into the bloodstream^32,33^. As such, these markers provide a clear indication of possible toxicity effects of OP-SBs accumulation on kidney, liver and hematological system, respectively. Analyses of the markers were performed at the 1 week and 1 month post treatment to determine the possible presence of acute and late toxicity, respectively. No significant difference was observed in the ALP/ALT activity, BUN and PF4 levels between the mice injected with 25 mg/kg OP-SBs and control, at both 1 week and 1 month post injection (Supplementary Fig. 17). PF4 levels remained low at <2.5 ng/mL at both 1 week and 1 month post injection of 50 mg/kg OP-SBs. A marginal but non-significant increase in ALT activity from 25.6 U/L in controls to 33.1 U/L in OP-SBs injected mice was observed at 1 month post injection (*p* = 0.289). When dose was further increased to 50 mg/kg OP-SBs, ALP and ALT activity remained comparable to controls at both 1 week post injection (Fig. 4a, b). We observed a significant increase in BUN levels compared to controls at 1 week post injection (32.9 mg/dL vs. 25.0 mg/dL, *p* = 0.001, Fig. 4c). Nonetheless, the BUN levels returned to 23.8 mg/dL at 1 month post injection (*P* = 0.830), reflecting a transient inhibition of kidney function without long-term toxicity. The above results were comparable with other similar studies^34,35^.

The comparable levels of all markers between control and 25 mg/kg dose up to 1 month post treatment suggested that the administration of OP-SBs at this dose was well tolerated and safe. Even with the increase to a higher dose of 50 mg/kg, the levels still remained comparable between treated and control mice, except for a slight transient inhibition of the kidney function. The high biocompatibility of the OP-SBs used in our study was consistent with previous studies on other silica-based nanoparticles^34,35^.

We further investigated the immunogenicity of the OP-SBs by monitoring the concentration of complement component 3 (C3) protein in the mouse serum post-treatment as shown in Supplementary Fig. 18. Activation of the complement system, a part of the innate immune system, results in consumption of C3^36^. As such, measurement of C3 concentration in serum could be used as one of the markers of nanoparticle immunogenicity. At 1 week post injection of the OP-SBs, C3 concentrations between healthy control mice and mice dosed at 25 mg/kg were comparable (17.06 ± 4.09 ng/mL vs. 14.22 ± 2.48 ng/mL, respectively, *P* = 0.361). However, a slight reduction in C3 concentration was observed with 50 mg/kg dose (10.70 ± 0.81 ng/mL) compared to healthy control (*P* = 0.0575), indicating some level of complement activation. Nonetheless, this effect was transient, with C3 concentration returning to normal by 1 month post injection of OP-SBs, indicating that there was no chronic complement activation induced by the OP-SBs treatment.

Activation of the complement system produces complement proteins which can bind to the surface of nanoparticles. This results in opsonization of nanoparticles, in turn enhancing recognition by phagocytic cells. Therefore, clearance of OP-SBs from the bloodstream by spleen macrophages and liver Kupffer cells, part of the MPS, would be enhanced by complement activation and nanoparticle opsonisation. This likely contributed to the aforementioned accumulation of OP-SBs primarily in the liver and spleen. In addition, the normalisation of C3 concentration by 1 month post injection implied that the complement system was no longer activated, despite the moderate accumulation of the OP-SBs in the liver and spleen.

Histopathological analysis of sample tissues in the major organs was performed to investigate for pathological changes in tissue morphology. The hematoxylin and eosin (H&E) stained tissue sections of the kidney, spleen, liver, heart, and lung were obtained from mice intravenously injected with 0 (control), 25 and 50 mg/kg OP-SBs, at 1 week (Figs. 4d) and 1 month (Fig. 4e) post injection. The H&E staining revealed that the histological features in these major organs tissues between the different treatment doses at different time-points were largely similar with no apparent abnormal features observed compared to samples from healthy control. Taken together, our hematological and histopathological results support the good biocompatibility of the OP-SBs, and that these nano-compounds were well tolerated in vivo.

### Photoactivation of therapeutic processes in vivo

Cal-27 tumors were grown subcutaneously in nude Balb/C mice and OP-SBs@azo-Psi were injected intratumorally and irradiated with a 980/808 nm NIR laser at specified time points (subsequent activation) to activate endosomal escape, siRNA release and ^1^O_2_ production (Group 4). As controls, Group 3 received intratumoral injection of Control-SBs@azo-Psi with 980 nm NIR irradiations at specified time points (simultaneous activation), Group 2 received intratumoral injection of OP-SBs@azo-Psi without subsequent NIR irradiation and Group 1 received intratumoral injection of saline. At the end of the study, mice in Group 4 showed significant reduction (>78%) in tumor growth in comparison to Group 2 (*p* < 0.01, *p* = 0.0008) and Group 1 controls (*p* < 0.01, *p* = 0.00001) as shown in Fig. 4f. There was also significant tumor reduction in comparison to Group 3 (*p* < 0.05, *p* = 0.0301) which was the conventional way of activating therapeutics simultaneously. The enhanced efficacy proves that subsequent programmed photoactivation indeed translates to better therapeutic outcomes. Group 3 showed significant tumor reduction in comparison to Group 2 (*p* < 0.05, *p* = 0.0141) and Group 1 (*p* < 0.01, *p* = 0.0003) since PDT is a commonly used, effective therapeutic modality. Group 1 and Group 2 did not show any significant differences in their tumor volumes (*p* < 0.05, *p* = 0.3167). There was no significant difference in body weight of Groups 1, 2, and 4 as seen from Fig. 4g. However, there was a slight but statistically significant (*p* < 0.01, *p* = 0.0032) reduction in the body weight of the mice in Group 3. This could possibly be due to the prolonged exposure to 980 nm NIR laser. Furthermore, the uptake and retention of the OP-SBs@azo-Psi in the tumor was studied by harvesting the tumor at specific time points and analyzing the sections by confocal microscopy. As seen from the Fig. 4h, i, it can be seen that the OP-SBs@azo-Psi are well distributed in the tumor and retained until PDT is performed (at 8 and 24 h post injection, respectively).

## Discussion

Hence, we have shown a very simple and user-friendly method for synthesizing OP-SBs in a versatile and modular fashion with modifiable properties like size, shape, excitation/emission wavelengths and luminescence intensity, by placing lanthanide activator/emitter ions in different regions of the SBs instead of in epitaxially grown shells. The orthogonal activation pattern using OP-SBs@azo-Psi was more effective than traditional non-orthogonal activation and increased the efficiency of PDT significantly in vitro and in vivo. The developed nanoplatform is not only restricted to PDT but allows a great deal of flexibility to be used for a wide range of applications requiring programmed photoactivation such as bi-directional optogenetics, light-based therapies, and synthetic biology.

## Methods

### Materials

Cyclohexane, Sodium dodecyl sulfate (SDS), Cetyltrimethylammonium bromide (CTAB), YCl_3_•6H_2_O (99.9%), YbCl_3_•6H_2_O (99.9%), ErCl_3_•6H_2_O (99.9%), GdCl_3_•6H_2_O (99.9%), NaOH (98+%), NH_4_F (98+%), 1-octadecene (90%), oleic acid (OA) (90%), (3-aminopropyl) triethoxysilane (APTES) (99%), tetraethyl orthosilicate (TEOS) (98%), NH_4_NO_3_ (98+%), 4-(Phenylazo)benzoyl chloride (97%), Zinc phthalocyanine, triethylamine (99.5%) and dimethyl sulfoxide (99%) were purchased from Sigma-Aldrich and used as received without further purification. The CellMask™ green plasma membrane stain, Hoechst 33342, ^1^O_2_ Sensor Green (SOSG) and Image-iT ™ LIVE green ROS detection kit were purchased from Thermo Fisher Scientific. Human Superoxide Dismutase 1 siRNA was obtained from Dharmacon and SOD1 ELISA Kit was obtained from Abcam.

### Synthesis of UCNPs

NaREF_4_ (RE = Y, Gd, Yb, Tm, Er) nanocrystals were synthesized following a high temperature coprecipitation method. In a typical process, at the beginning, 1 mmol RECl_3_ aqueous solution with stoichiometric ratio was added in a trineck flask (100 mL). After the fully removal of water by elevating the temperature above the boiling point, the residuals were dissolved by adding 15 mL of 1-octadecene and 6 mL of oleic acid into the flask and heating the mixture to 156 °C. The mixture was maintained at 156 °C for 10 mins to allow the complete formation of RE-oleate complexes. The resulting solution was then cooled down to room temperature and mixed with a methanol solution (5 mL) containing NH_4_F (4 mmol) and NaOH (2.5 mmol). To remove the methanol from the system, the mixture was raised to 120 °C for 10 mins. Then the solution was degassed for 15 mins to fully remove residual methanol and oxygen. Subsequently, the resulting solution was raised to 300 °C for 1 h under argon environment. The products were precipitated down with acetone, under centrifugation at 7370 *× g* for 10 mins, washed with acetone, and finally dispersed in 20 mL of cyclohexane for further use.

Core-shell and core-shell-shell UCNPs were prepared through epitaxial growth. The as-prepared core NaREF_4_ nanocrystals were used as seeds for epitaxial shell growth. In a typical process, based on the core-shell ratio, certain amounts of aqueous solution of RECl_3_ (RE = Y, Gd, Yb, Tm, Er, Nd) were added into a 100 mL flask. After the fully removal of water by elevating the temperature above the boiling point, the residuals were dissolved by adding 15 mL of 1-octadecene and 6 mL of oleic acid into the flask and heating the mixture to 156 °C. The mixture was maintained at 156 °C for 10 mins to allow the complete formation of RE-oleate complexes. Upon cooling of the RE-oleate precursors to room temperature, the as-prepared core nanoparticles dispersed in 20 mL of cyclohexane were added, and the resulting mixture was then heated at 120 °C for 20 mins to evaporate the cyclohexane. Subsequently, the solution was cooled to room temperature, followed by the addition of a methanol solution containing NH_4_F and NaOH, the overall amount of methanol is based on the RECl_3_ precursor added, 5 mL methanol solution containing 4 mmol NH_4_F and 2.5 mmol NaOH is required for every 1 mmol RECl_3_ precursor added. The resulting mixture was vigorously stirred and then heated at 120 °C for 10 mins. After that, the reaction was degassed for 10 mins to evaporate the residual methanol and oxygen in the solution. Finally, the temperature was raised to 300 °C and kept under argon atmosphere for 1.5 h. The resultant nanoparticles were precipitated down after the addition of acetone under 7370* × g* centrifugation for 10 mins, washed with acetone, dispersed in 20 mL cyclohexane for further usage.

### Synthesis of OP-SBs

One mililiter mixed UCNPs cyclohexane solution (5 mg/mL) was added to 10 mL water containing 7 mg SDS. Then the mixture was vortexed vigorously and sonicated to form an emulsion. Finally, after the evaporation of the low boiling-point cyclohexane at 70 °C with strong stirring (230* × g*) for 3 h, OP-SBs were obtained and dispersed in water.

### Synthesis of mesoporous silica-coated OP-SBs

Mesoporous silica-coated OP-SBs (OP-SBs@mSiO_2_) were synthesized by a multistep process. Ten microliter of APTES was added to 10 mL of OP-SBs solution (the concentration of UCNPs is 0.5 mg/mL), and the solution was stirred for 30 mins to let the OP-SBs adsorb positively-charged APTES on their surface. After that, 10 mL water, containing 35 mg CTAB and 2 mL ethanol were added to the above solution. After stirring for 30 mins, 300 µL of 33% ammonium hydroxide solution was added into the mixture. Then 75 µL of TEOS was added dropwise into the above solution and kept string for 1 day. After washing twice with acetone and methanol mixture (volume ratio of 1:1), the OP-SBs with silica coating dispersed in 10 mL ethanol and mixed with 10 mL ethanol containing 300 mg NH_4_NO_3_. The solution was refluxed at 60 °C for 2 h to remove the CTAB to get mesoporous silica coating. The obtained samples were then washed by acetone and ethanol mixture twice and dispersed in 10 mL of ethanol for further use.

### Azobenzene modification of OP-SBs

The OP-SBs were modified with *N*-(3-triethoxysilyl)propyl-4-phenylazobenzamide (azobenzene) to obtain OP-SBs@azo. First, the azobenzene solution was prepared by adding 3-Aminopropyltriethoxysilane (1.22 g, 5.5 mmol) and triethylamine (0.556 g, 5.5 mmol) into 50 mL of the ethanol solution of 4-phenylazobenzoyl chloride (1.22 g, 5 mmol). The solution was stirred for 12 h under argon atmosphere at room temperature and followed by purification via filtration. The resulting azobenzene was further concentrated to a solid form using a rotary evaporator. The as-prepared azobenzene (0.08 g) was dispersed in ethanol and added drop-wise to the ethanol solution of OP-SBs (10 mg). The suspension was stirred for 1 h at 80 °C, and the OP-SBs@azo were obtained by centrifugation at 16580* × g* for 20 mins and resuspended in water for subsequent studies. The modification was characterized by UV-vis spectrophotometry.

### ZnPC and siRNA loading onto OP-SBs@azo

The photosensitizer, ZnPC and siRNA are loaded on to the mesoporous silica by physical adsorption and electrostatic interactions^37,38^. For the loading of ZnPC, 0.2 mg of ZnPC was dispersed in 1 mL solution of mesoporous silica-coated OP-SBs@azo in DMSO. The solution was kept for shaking at room temperature for 4 h followed by collection of OP-SBs@azo-P (OP-SBs@azo loaded with photosensitizer) via centrifugation at 16580* × g* for 20 mins. For siRNA loading, OP-SBs@azo-P was centrifuged at 16580* × g* for 20 mins and the supernatant was discarded.nIn all, 1 µM of siRNA dispersed in siRNA buffer (60 mM HCl, 6 mM HEPES, and 0.2 mM MgCl_2_) was added to 1 mg of the OP-SBs@azo-P pellet. The solution was stirred for 4 h at 110* × g* and irradiated with 808 nm for 15 mins every 2 h to enhance the loading of siRNA. Finally, the OP-SBs@azo-Psi (OP-SBs loaded with photosensitizer and siRNA) were centrifuged at 16580* × g* for 20 mins and the supernatant was discarded. The loaded nanoparticles were re-dispersed in water and stored at 4 °C for further use. To confirm loading, the supernatant of OP-SBs solution was collected before and after the addition of ZnPC and siRNA and analysed by absorbance and fluorescence spectrophotometry respectively.

### Stability of OP-SBs@azo-Psi

To evaluate the stability of the OP-SBs@azo-Psi, the nanoparticles were dispersed in water and 10% FBS. The hydrodynamic size distribution of OP-SBs@azo-Psi were recorded using Malvern zetasizer at regular intervals for 72 h.

### Cell and spheroid culture

HeLa and Cal27 cells were procured from American Type Culture Collection (ATCC) and grown in DMEM and RPMI (Life Technologies) culture medium, respectively, supplemented with 10% FBS (Life Technologies), 100 units/mL of penicillin, and 100 μg/mL of streptomycin. The cell lines were tested for mycoplasma contamination with MycoAlert^TM^ Mycoplasma Detection Kit (Lonza) prior to use. 3D tumour spheroids were prepared by seeding cells in an ultra-low attachment 96-well microplates (Sigma) and growing them in DMEM and RPMI culture media (Life Technologies), respectively, supplemented with 10% FBS (Life Technologies), 100 units/mL of penicillin, and 100 μg/mL of streptomycin. The 2D cells and 3D spheroids were maintained at 37 °C in a humidified, 5% (v/v) carbon dioxide (CO_2_) atmosphere.

### Cell viability assay

Cell viability assay was investigated for different concentrations of OP-SBs@azo-Psi as well as for different excitation wavelengths (phototoxicity). HeLa cells were seeded in 96-well plates for 2D culture and in ultra-low attachment 96-well plates for 3D spheres. Both the plates were maintained at 37 °C in the CO_2_ incubator. After incubation of 24 h (for 2D culture) and 72 h (for 3D spheres) of incubations, the plates were either treated with OP-SBs@azo-Psi at different concentrations (0–1 mg/mL) or subjected to 980 and 808 nm NIR irradiations for timings relevant to the study. After 24 h of incubation, the cell viability was evaluated using MTS assay (for 2D culture) and ATPlite assay (for 3D culture), as per the manufacture’s protocol.

### Cellular uptake of OP-SBs@azo-Psi

HeLa cells were incubated with 0.5 mg/mL of OP-SBs@azo-Psi and kept at 37 °C in humidfied CO_2_ incubator for different time points. At the end of each incubation time, the cells were washed thrice to get rid of the excess OP-SBs@azo-Psi in the media and on the cells surfaces. The washed cells were trypsinized by 0.05% trypsin in 1x PBS and collected for measuring the luminescence emission of OP-SBs@azo-Psi under 980 nm excitation using NIR spectrophotometer. To explore the mechanism of OP-SBs@azo-Psi uptake, inhibitors-based blocking of the uptake pathways was carried out. HeLa cells were incubated with OP-SBs@azo-Psi and different inhibitors such as chlorpromazine, cytochalasin B, nystatin and filipin to block endocytosis, macropinocytosis and lipid raft-mediated uptake^39^. After incubation, the cells were processed as mentioned before to record the luminescence emission.

The uptake was also investigated visually by counterstaining the OP-SBs@azo-Psi-treated cells and 3D spheroids with CellMask™ green plasma membrane stain and Hoechst 33342. The excess OP-SBs@azo-Psi-after treatment was removed by washing cells and spheroids with 1x PBS, followed by the addition of fresh culture media. To this, 1x HBSS solution of CellMask™ green plasma membrane stain (for cell membrane) was added and incubated in the CO_2_ incubator. After 5 mins, Hoechst 33342 (for cell nucleus) was added at a final concentration of 1.0 μM and the cells and spheroids were incubated for another 5 mins. After incubation, the cells and spheroids were washed thrice with 1x PBS to get rid of excess stains and resuspended in culture medium prior to imaging.

### Singlet oxygen release and detection

Release of ^1^O_2_ in solution was detected by ^1^O_2_ sensor green (SOSG). 2 mL of OP-SBs@azo-Psi (1 mg/mL) were taken in a cuvette and 4 µL of SOSG solution in methanol was added and mixed by vortexing. The suspension was excited with 980 nm (2.5 W/cm^2^) continuous wave laser and the luminescence intensity was recorded (ex/em = 490/515) at 0, 15, 30, 45, and 60 mins to evaluate the temporal control of ^1^O_2_ production by 980 nm-mediated OP-SBs@azo-Psi activation. To further investigate the ^1^O_2_ release, the OP-SBs@azo-Psi sample mixed with SOSG was irradiated using 980 nm laser for 1 h and stored at 4 °C for 18 h, after which they were irradiated for 1 h to check the capability of the same sample to produce ^1^O_2_ at different time points. After irradiation, the luminescence intensity was recorded at 0 and 18 h time points, each at 0, 15, 30, 45, 60 mins. Detection of ^1^O_2_ was also performed for release under 808 nm excitation following the same steps as elucidated above and compared to that under 980 nm excitation.

The ^1^O_2_ detection in living cells of 2D culture and 3D spheres was performed using Image-iT Green Live ROS Detection Kit. HeLa cells were treated with 0.5 mg/mL OP-SBs@azo-Psi and incubated for 8 h. The cells and spheroids were then washed thrice with 1x PBS to get rid of the excess OP-SBs@azo-Psi and the ROS produced was detected using the manufacturer’s protocol provided with the kit. For counter staining, HOECHST 33342 was added and incubated for 5 mins. Prior to imaging, the excess stain was washed off and the cells were resuspended in culture media.

### ELISA for gene knockdown

Gene knockdown was studied using Human Superoxide Dismutase 1 ELISA Kit. HeLa cells were treated with OP-SBs@azo-Psi (0.5 mg/mL). They were further irradiated with different lasers (980 or 808 nm) and 48 h after incubation, the cells were lysed using cell extraction buffer and ELISA was carried out based on the manufacturer’s protocol provided with the kit.

### siRNA release and detection

OP-SBs@azo-Psi in water was taken at a concentration of 1 mg/mL and subjected to 808 nm NIR irradiation (2.5 W/cm^2^) for release of siRNA. The fluorescence intensity was then recorded at regular intervals (0, 15, 30, 45, 60 mins) to assess the temporal control of siRNA release by 808 nm-mediated OP-SBs excitation.

For release in live cells, HeLa cells were incubated with Cy3-labelled OP-SBs@azo-Psi. After overnight incubation, the cells were subjected to 808 nm irradiation. The siRNA release in cells was imaged using fluorescence microscopy and quantified using flow cytometry. Hoechst 33342 was used as counterstain for imaging. For flow cytometry, the cells were trypsinized and filtered through a 40 μm cell strainer to get a single cell suspension. The cells were then centrifuged and re-suspended at a density of 1 × 10^6^ cells/mL in pre-warmed RPMI-1640 medium containing 2% FBS and analysed using Cytoflex LX (Beckman Coulter).

### Programmed activation

OP-SBs@azo-Psi were subjected to different durations of 808 and 980 nm irradiations (808:980 nm = 60:0, 45:15, 30:30, 15:45, 0:60 mins, 2.5 W/cm^2^). After irradiation, the OP-SBs@azo-Psi were centrifuged at 16580* × g* for 15 mins and the luminescence intensity of SOSG and siRNA was recorded in the supernatant to evaluate the programmed activation of ^1^O_2_ production and siRNA release from the OP-SBs@azo-Psi respectively.

### Simultaneous and subsequent activation

For simultaneous (non-orthogonal) activation, Control-SBs@azo-Psi that were only activatable by 980 nm excitation were synthesized. The subsequent (orthogonal) activation was achieved using the OP-SBs@azo-Psi excitable by both 980 and 808 nm radiations. After irradiating the OP-SBs@azo-Psi and Control-SBs@azo-Psi, the detection of siRNA release and ^1^O_2_ production was performed by the methods described earlier.

### Effect of PCI on PDT and gene knockdown

Cancer cell killing and SOD1 knockdown was studied with and without PCI. HeLa and Cal27 2D cells and 3D spheroids were incubated with OP-SBs@azo-Psi and subjected to PDT (60 mins of 980 nm NIR irradiation, 2.5 W/cm^2^) with and without PCI (15 mins of 980 nm NIR irradiation, 2.5 W/cm^2^). The cell viability in 2D cells and 3D spheroids was assessed by MTS assay and ATPlite assay, respectively, performed based on the manufacturer’s protocol. For siRNA release, OP-SBs@azo-Psi incubated HeLa cells were irradiated with NIR laser for siRNA release (30 mins of 808 nm NIR irradiation, 2.5 W/cm^2^) with and without PCI (15 mins of 980 nm NIR irradiation, 2.5 W/cm^2^). The gene expression was then evaluated by SOD1 ELISA kit as described earlier.

### Simultaneous vs subsequent activation

Cells and spheroids were incubated with OP-SBs@azo-Psi and Control-SBs@azo-Psi. The Control-SBs@azo-Psi group was irradiated as follows: 8 h @980 nm, 8.5 h @980 nm, 26 h @ 980 nm irradiation and the OP-SBs@azo-Psi group was irradiated as follows: 8 h @980 nm, 8.5 h @808 nm, 26 h @980 nm, 2.5 W/cm^2^. After NIR irradiations, the cells were incubated for another 48 h and the cell viability was assessed as described earlier.

### Biodistribution of OP-SBs in vivo

All procedures performed were approved by the Institutional Animal Care and Use Committee (IACUC, 2016/SHS/1150) Singapore Health Services Pte Ltd. Balb/c nude mice aged 5–6 weeks were intravenously injected with OP-SBs via tail vein injection at a dose of 25 and 50 mg/kg and sacrificed by cervical dislocation at 6 h, 24 h, 1 week, and 1 month post injection (*n* = 5 mice per time point per dose). The liver, spleen, heart, lung, kidney, blood, urine and faeces were harvested and weighed. The tissues were solubilised and homogenised by adding 1 mL of Solvable tissue solubiliser (PerkinElmer, U.S) and heated in a 50 °C water bath for 2 h with constant agitation. The solubilised tissues were then mixed with 300 µL of aqua regia each and the mixture was left to stand for 1 h at room temperature. The resulting mixture was diluted to 5 mL with UltraPure water (Invitrogen, Singapore) and inductively coupled plasma mass spectrometry was performed to determine the mass concentration of Ytterbium (Yb) and Yttrium (Y). The total mass of Yb and Y in an organ was then calculated by multiplying the Yb and Y concentration with the mass of the respective organ. The percentage of injected Yb and Y dose in the organ was then calculated using the total mass of Yb and Y.

### Hematological analysis

To analyse the function of the major organs and the biocompatibility of the OP-SBs, balb/c nude mice were intravenously injected with OP-SBs of dose 0 (control), 25 and 50 mg/kg. Blood was drawn at 1 week and 1 month post injection via cardiac puncture upon sacrifice of the mice. The blood was allowed to clot at room temperature, before centrifuging at 11510* × g* at 4 °C for 10 mins to obtain the serum. Liver function was determined by measuring the activity levels of ALP and ALT using Alkaline Phosphatase Assay Kit (Abcam, ab83369) and ALT Assay Kit (Abcam, ab241035), respectively. Kidney function was determined by measuring serum blood urea nitrogen (BUN) using Urea Assay Kit ll (Abcam, ab234052). Blood compatibility was determined by serum level of Platelet Factor 4 (PF4) using PF4 (CXCL4) Mouse Simple Step ELISA Kit (Abcam, ab202403). Complement system activity was determined by measuring the concentration of Complement C3 protein in serum using Complement C3 Mouse ELISA Kit (Abcam, ab157711).

### Histopathological analysis of major organs

To determine the acute and chronic effect of the OP-SBs accumulation on tissue health, balb/c nude mice were intravenously injected with OP-SBs of dose 0 (control), 25 and 50 mg/kg. Liver, spleen, heart, lung and kidney tissue were harvested at 1 week and 1 month post injection. The tissues were fixed in neutral-buffered formaldehyde (10%) for 24 h. The fixed tissue was then dehydrated in increasing concentrations of ethanol and immersed in NeoClear Xylene substitute, followed by immersion in paraffin. The tissues were then sectioned into 5 μm tissue sections using a microtome (Leica, Germany) and stained with hematoxylin and eosin (H&E). Bright-field images were obtained using a Nikon Eclipse 80i upright microscope (Nikon, Japan) with a 20x objective and equipped with a DS-Ri2 camera (Nikon, Japan).

### In vivo PDT

This study conforms to the Guide for the Care and Use of Laboratory Animals published by the National Institutes of Health, USA and protocol approved by the Institutional Animal Care and Use Committee (IACUC), National University of Singapore. Tumors were developed in Balb/C nude mice (5–6 weeks old) by subcutaneously injecting 10^7^ Cal-27 oral carcinoma cells suspended in 100 µL of matrigel (Corning, cat. no. 354234) in the lower right flank. 2–3 weeks after inoculation of tumor cells, the mice were randomly divided into different groups. These groups were: Group 4- OP-SBs@azo-Psi + 980/808 nm NIR (subsequent activation), Group 3- Control-SBs@azo-Psi + 980 nm NIR (simultaneous activation), Group 2- OP-SBs@azo-Psi without NIR irradiation, and Group 1-saline injected. At this stage, the tumors were ~4–7 mm in diameter (Day 1 of measurements, 3 weeks post inoculation). The tumor sizes were measured for all mice and recorded. At day 3, Groups 1, 2, and 3 were injected with 50 mg/kg of respective OP-SBs. After 8 h of injection, Groups 3 and 4 were exposed to 980 nm NIR light (5 min, 0.6 W/cm^2^). After a 30 min gap to allow for PCI to occur, the tumors were irradiated (8 min, 0.6 W/cm^2^) with either 808 nm (Group 4) or 980 nm (Group 3) light to release anti-SOD1 RNA. At 26 h post injection, mice in groups 3 and 4 were irradiated with 980 nm light (20 min, 0.6 W/cm^2^) to trigger PDT. For all 4 groups, mice were monitored regularly over 9 days, at the end of which, the mice were sacrificed using carbon dioxide overdose. Tumor size and body weight was measured thrice a week. The tumor volume, V, was calculated using the formula *V* = (W^2^xH)/2 as reported previously, where H is the tumor height and W is the perpendicular width to H. Tumor sizes were normalized to the tumor size at day 1 and plotted against time to indicate change in tumor volume over time. The body weight of the mice in all the groups were monitored regularly over the study period.

### OP-SBs uptake in tumor tissue

To study the distribution of the OP-SBs in the tumor at the relevant therapy time points (8 and 26 h post injection), tumors injected with these particles were excised at these time points and snap frozen using liquid nitrogen and isopentane. They were cryosectioned at a thickness of 10 µm onto PLL-coated slides, fixed using 4% paraformaldehyde and counter-stained with DAPI. Samples were imaged using a fluorescence confocal microscope (Nikon C1 Confocal, Nikon, Tokyo, Japan) for OP-SBs distribution (excitation 980 nm, emission 650 nm). The nuclei were imaged via DAPI staining (excitation 360 nm, emission 470 nm).

### Statistical analysis

The normality of the populations was initially tested. The mean values of the different treatment groups were then statistically compared to that of the control group using ANOVA for normally distributed populations and Kruskal-Wallis ANOVA for populations that were not normally distributed using OriginPro 8.1. *P* < 0.05 was considered statistically significant.

### Reporting summary

Further information on research design is available in the Nature Research Reporting Summary linked to this article.

## Supplementary information

Supplementary Information

Reporting Summary

## Data Availability

The data that support the findings of this study are available from the corresponding author upon reasonable request.
