# Supplementary material for: Upconversion superballs for programmable photoactivation of therapeutics
Source: Nat Commun. 2019 Oct 8;10:4586. doi: 10.1038/s41467-019-12506-w (PMC6783568; doi:10.1038/s41467-019-12506-w)
Supplement: Supplementary file 1 — Supplementary Information [file 41467_2019_12506_MOESM1_ESM.pdf]

SUPPLEMENTARY INFORMATION

Upconversion Superballs for Programmable Photoactivation of Therapeutics

Zhen Zhang[1#], Muthu Kumara Gnanasammandhan Jayakumar[1#], Xiang Zheng[1,2#], Swati Shikha[1], Yi Zhang[1], Akshaya Bansal[1], Dennis J.J Poon[3], Pek Lim Chu[4], Eugenia L.L Yeo[5], Melvin L.K. Chua[3,4,5], Soo Khee Chee[4,5,6] & Yong Zhang[1,2,*]

Supplementary Table 1. Abbreviations, structural and compositional details of UCNPs used in study

Name	Core	Shell	Shell	Core:Shell Ratio
A	$NaYF_4$:60%Yb, 20%Gd, 2%Er	$NaLuF_4$:25%Y	--	1:1
B	$NaYF_4$:30%Yb, 0.5%Tm	$NaYF_4$:10%Yb	$NaNdF_4$:10Yb	1:0.2:1
C	$NaYF_4$:20%Yb, 2%Er	$NaYF_4$:10%Yb	$NaNdF_4$:10Yb	1:0.2:2
D	$NaYF_4$:20%Yb, 2%Er	$NaLuF_4$:25%Y	--	1:1
E	$NaYF_4$:30%Yb, 0.5%Tm	$NaLuF_4$:25%Y	--	1:1

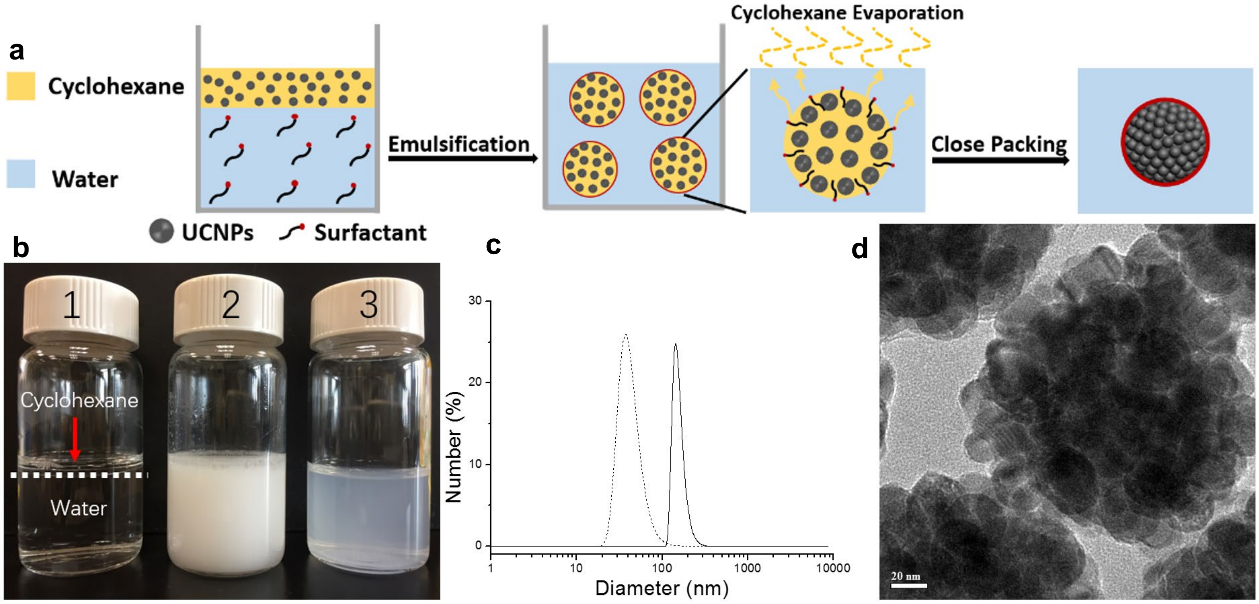

Supplementary Figure 1. Emulsion-based self-assembly of OP-SBs. **a,** A scheme showing the self-assembly of UCNPs into OP-SBs structures via an emulsion-based process. **b,** Photos of OP-SBs fabrication process: 1) 1 mL cyclohexane suspension of the mixed UCNPs A and B (total concentration: 5 mg/mL) mixed with 10 mL water containing 7 mg SDS, forming immiscible two layers when put together. 2) After sonication and vertexing, turbid microemulsion solution was obtained. 3) OP-SBs solution was obtained after cyclohexane evaporated form water. **c,** DLS characterization of mixed UCNPs cyclohexane suspension (dash line) and corresponding OP-SBs water suspensions (solid line). **d,** High resolution TEM image of OP-SBs.

The oil phase (cyclohexane) containing well-dispersed oleic acid (OA)-capped two types of UCNPs (5 mg/mL) and the aqueous phase containing surfactants (sodium dodecyl sulfate (SDS)) were mixed together at an oil-to-water ratio of 1:10 (v/v). A stable O/W emulsion system was obtained under vigorous stirring and sonication, by which the well-dispersed UCNPs were confined in the emulsion droplets that were stabilized by the surfactants present in the aqueous phase. Subsequently, the low-boiling solvent (cyclohexane) was evaporated from the oil emulsion droplets by heating the solution at 70 °C. During solvent removal, the droplets shrink and the UCNPs in the droplets get concentrated and pack closely with each other, thereby assembling to form OP-SBs. Through hydrophobic Van der Waals interactions, the SDS's alkane chains from the aqueous phase will interdigitate spontaneously with the OA's alkane chains located on the outer surface of the OP-SBs. Hence the obtained spherical OP-SBs were well-dispersed in water. Zeta potential characterization indicated that these OP-SBs had a negative surface charge (about -61 mV) due to the presence of the anionic surfactant (SDS) on their surfaces.

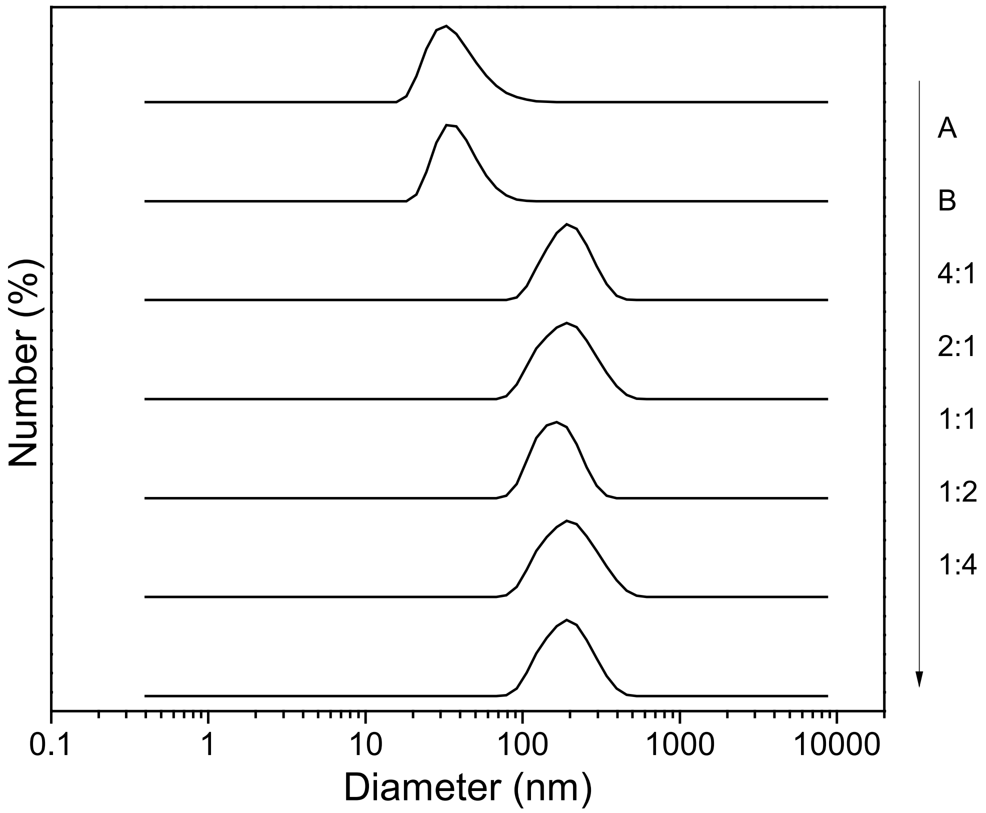

Supplementary Figure 2. DLS results of as prepared superball with different A:B mixing ratios.

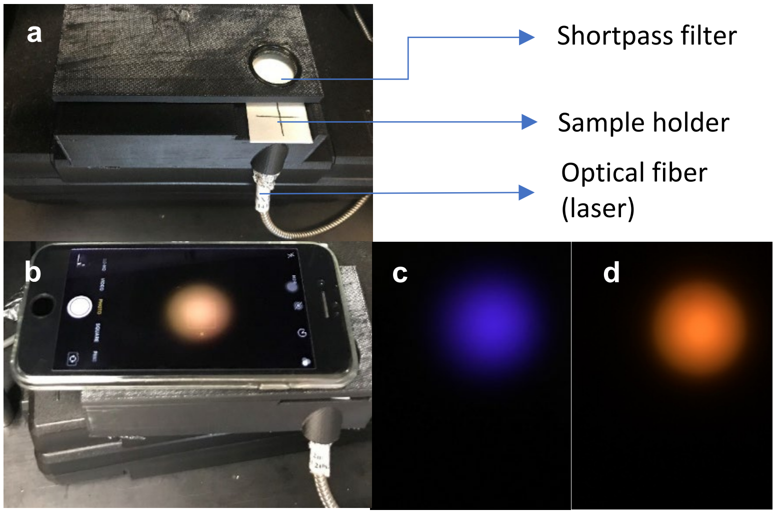

Supplementary Figure 3. Details of smartphone photography device: **a,** Device setup, **b,** Picture showing the working condition. Original camera photos of OP-SBs under 808 (**c**) and 980 nm (**d**) laser excitation.

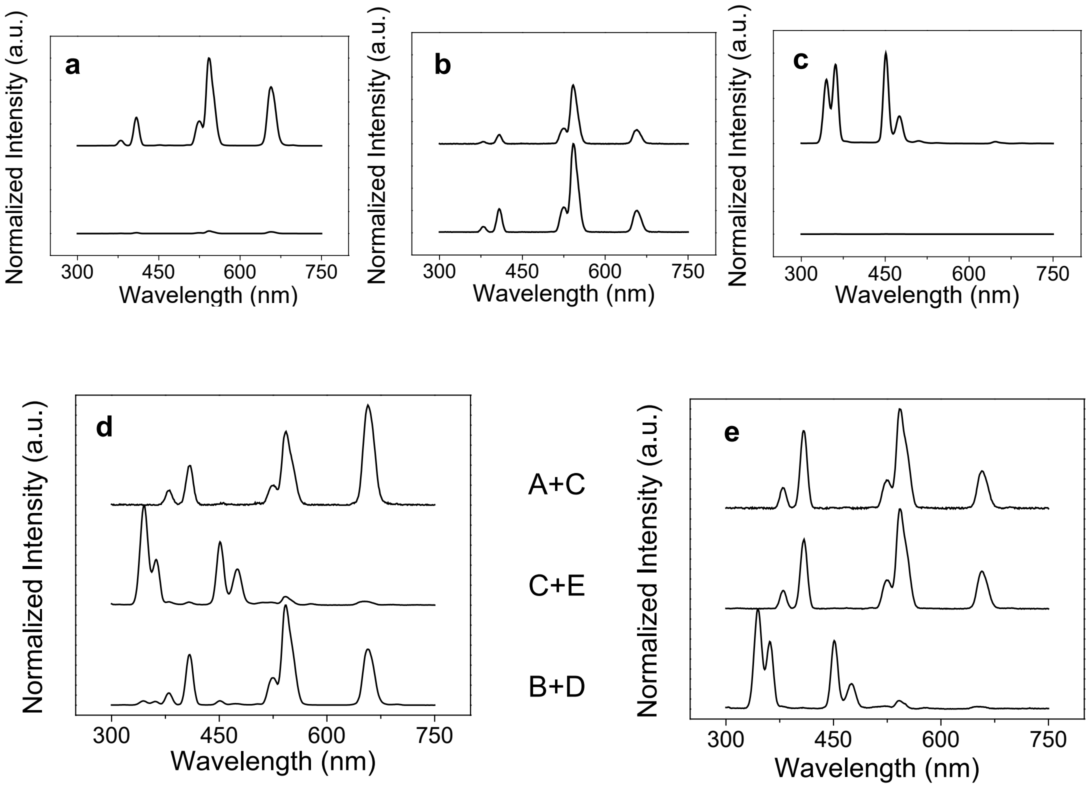

Supplementary Figure 4. a-c, Spectrums of UCNPs C, D and E under 980 nm laser (above) and 800 nm laser (bottom). Spectrums of OP-SBs suspension under 980 nm (**d**) and 800 nm laser (**e**).

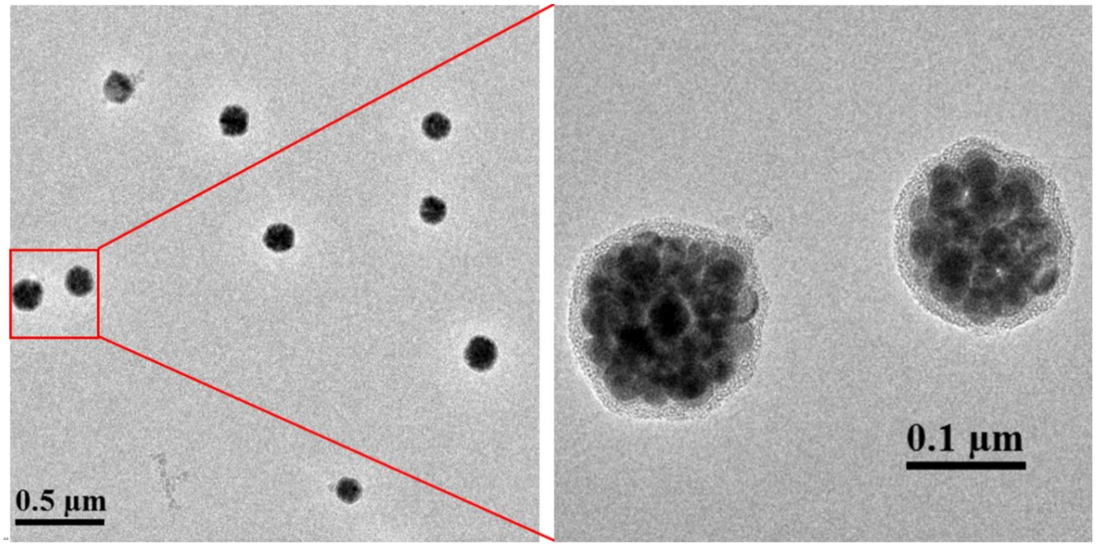

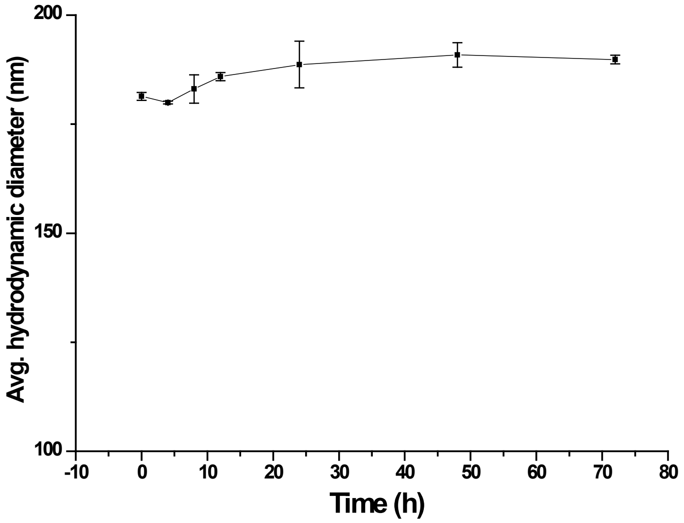

Supplementary Figure 5. TEM images of mesoporous silica coated OP-SBs (OP-SBs@mSiO_2) with inset magnifications and DLS data showing stability of OP-SBs@mSiO_2 in pH=4.5 water solution conducted over 72 hours. Error bars represent the standard deviation of three distinct measurements

Supplementary Table 2. Characteristics of OP-SBs@azo-Psi.

**Hydrodynamic radius**	**Zeta Potential**	**Loading content** (/mg UCNPs)	
232.1±14.4	-21.7 mV±0.41	101 μg ZnPc	8.4 μg siRNA

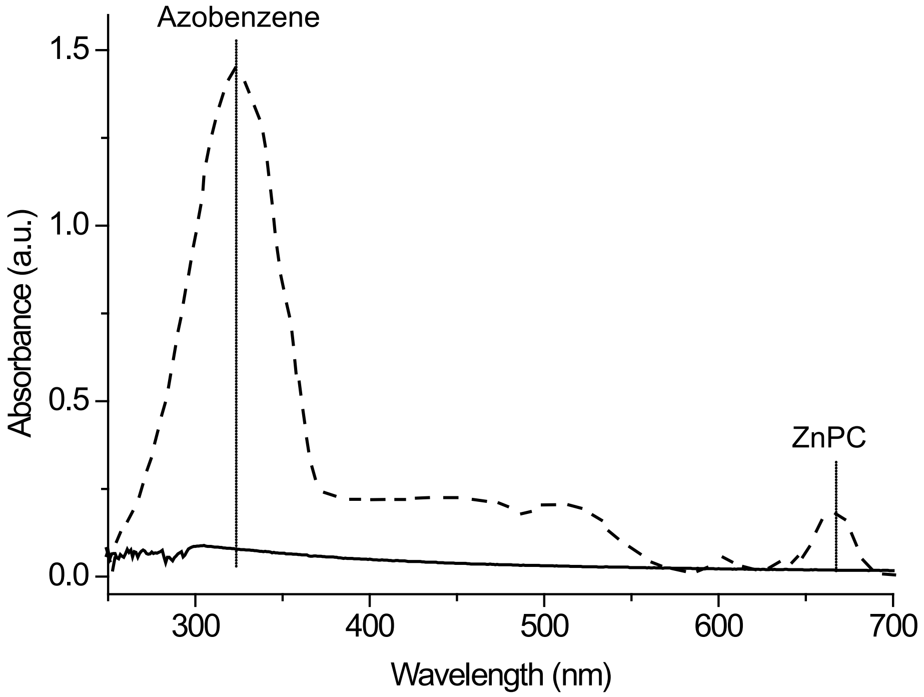

Supplementary Figure 6. UV-Vis absorbance spectrophotometry of OP-SBs@mSiO_2 (solid line) and OP-SBs@mSiO_2 after loading ZnPc and azobenzene (dash line). Absorbance peaks of azobenzene and ZnPC are specified in the graph (dot lines).

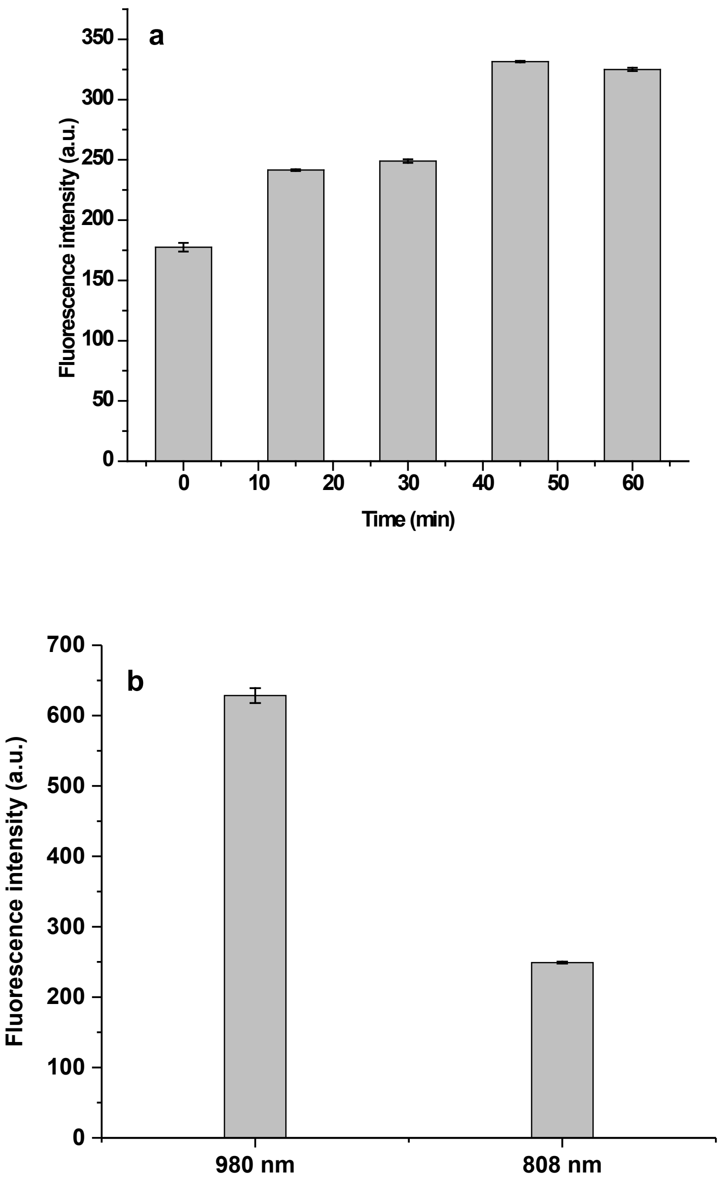

Supplementary Figure 7. Singlet oxygen production using 800 nm excitation of the OP-SBs@azo-Psi (**a**) and comparison with 980 nm excitation (**b**). Error bars represent the standard deviation of three distinct measurements

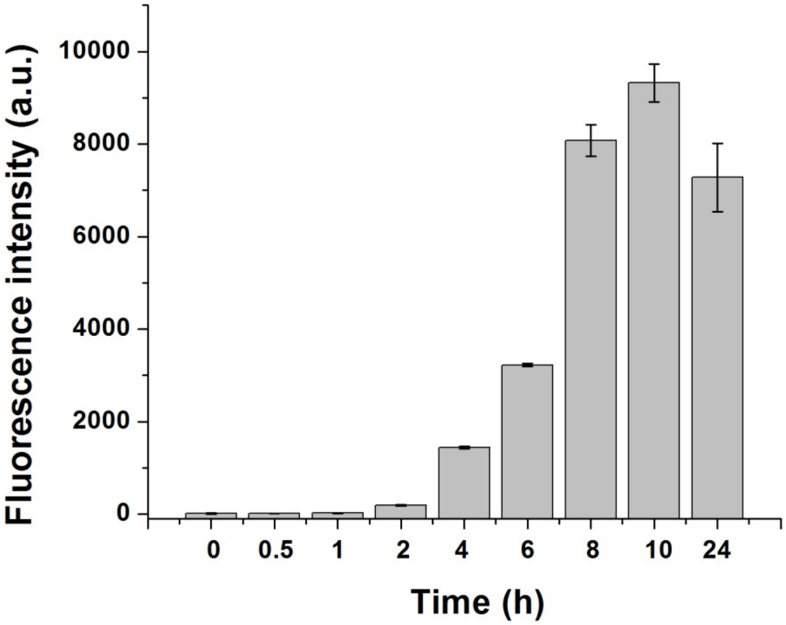

Supplementary Figure 8. Cellular uptake of OP-SBs over time. Error bars represent the standard deviation of three distinct measurements

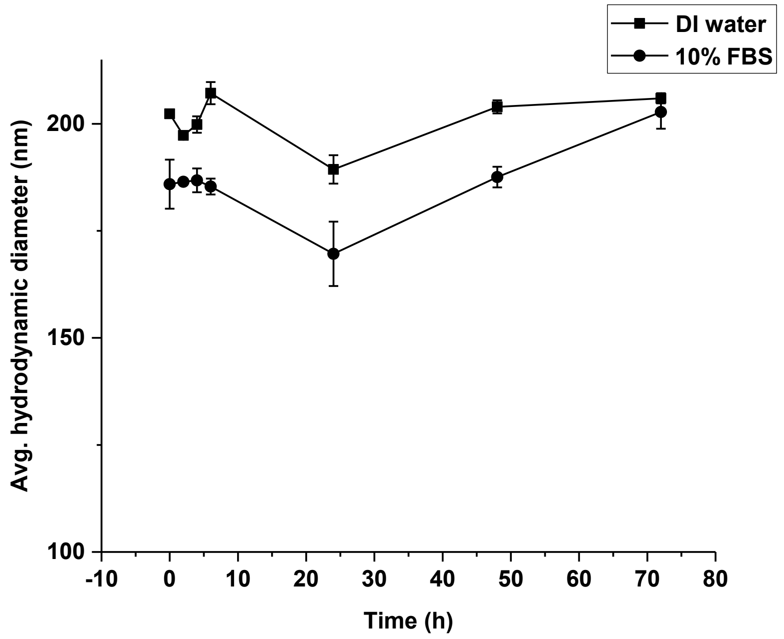

Supplementary Figure 9. DLS data showing stability of OP-SBs@azo-Psi in DI water and 10% FBS conducted over 72 hours. Error bars represent the standard deviation of three distinct measurements

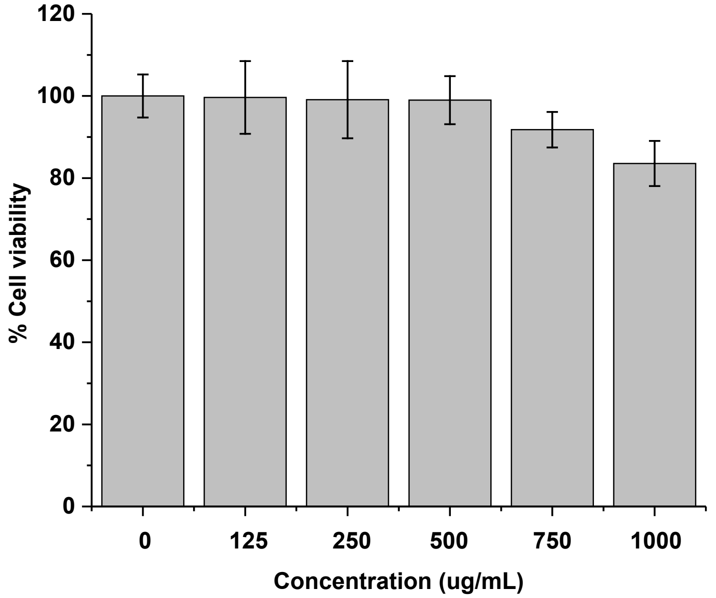

Supplementary Figure 10. Cytotoxicity of various concentrations of OP-SBs@azo-Psi on HeLa cells. Error bars represent the standard deviation of three distinct measurements

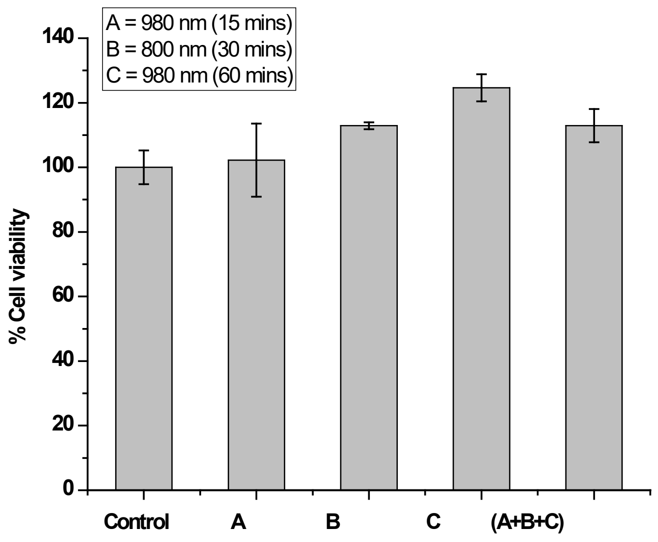

Supplementary Figure 11. Phototoxicity of NIR light on HeLa cells. Error bars represent the standard deviation of three distinct measurements

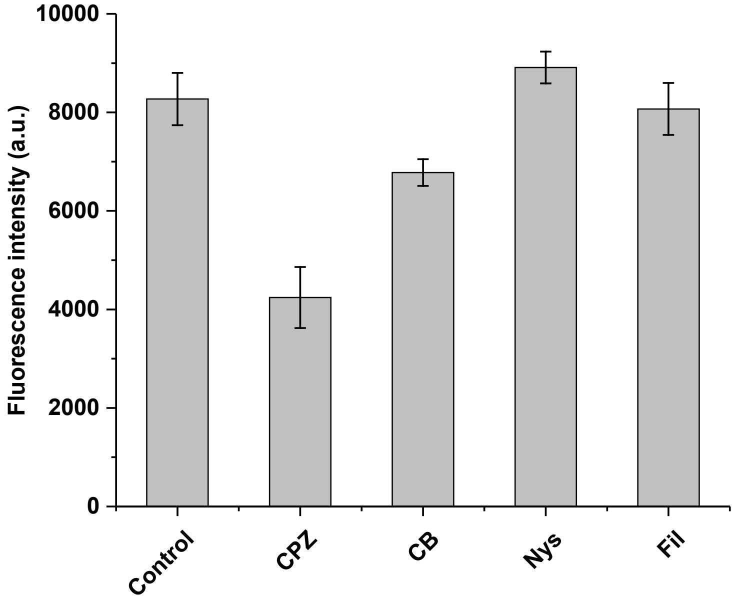

Supplementary Figure 12. Elucidation of the mechanism of OP-SBs@azo-Psi uptake using inhibitors of endocytosis, macropinocytosis and lipid raft mediated uptake. Error bars represent the standard deviation of three distinct measurements

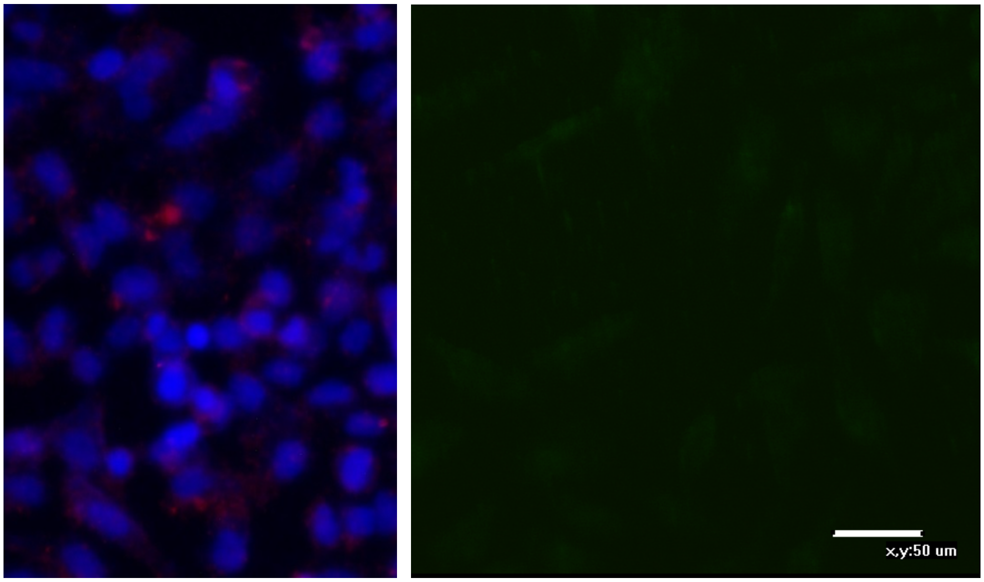

Supplementary Figure 13. siRNA release in the absence of NIR.

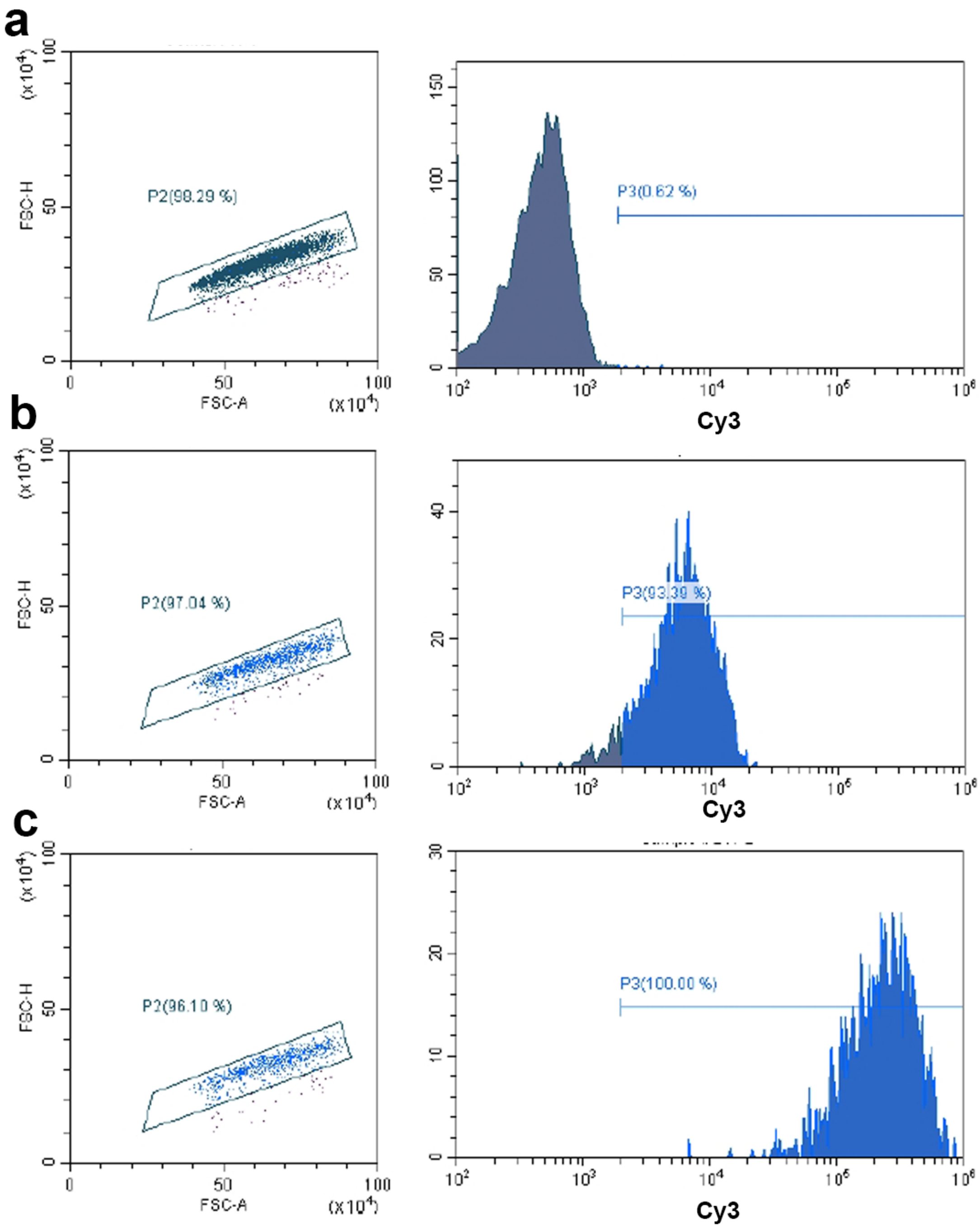

Supplementary Figure 14. Quantification of siRNA release in HeLa cells treated with OP-SBs@azo-Psi and excited with an 808 nm laser. (**a**: Control; **b**: OP-SBs@azo-Psi; **c**: OP-SBs@azo-Psi +NIR).

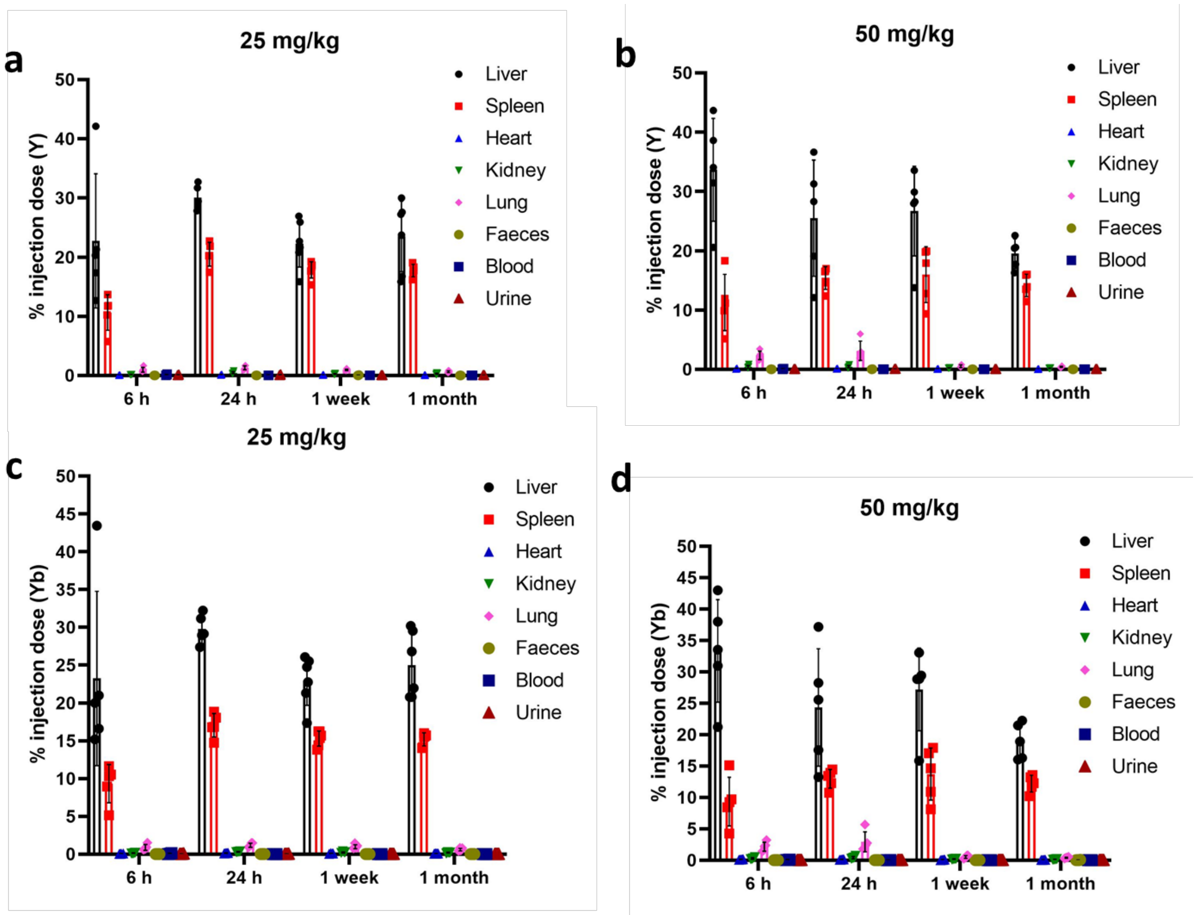

Supplementary Figure 15. Percentage of injected dose of Y (**a, b**) and Yb (**c, d**) in mice treated with 25 mg/kg OP-SBs (**a, c**) and 50 mg/kg OP-SBs (**b, d**). Error bars represent the standard deviation of five distinct measurements

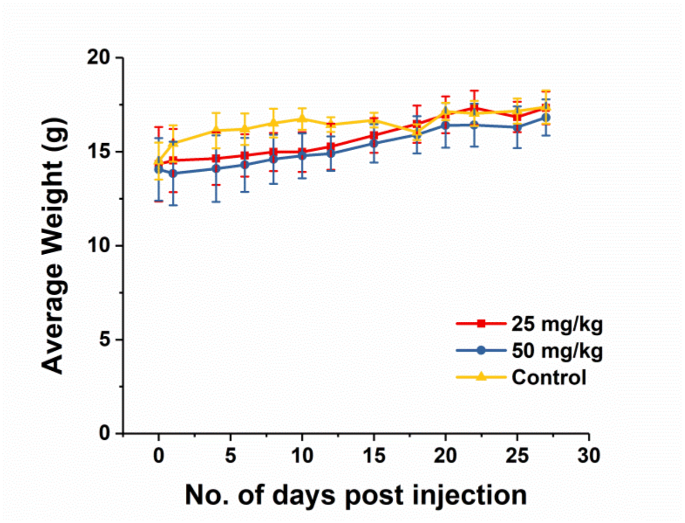

Supplementary Figure 16. Body weight of 3 groups were compared: untreated mice (control); mice injected with 25 mg/kg OP-SBs; mice injected with 50 mg/kg OP-SBs. Error bars represent the standard deviation of five distinct measurements.

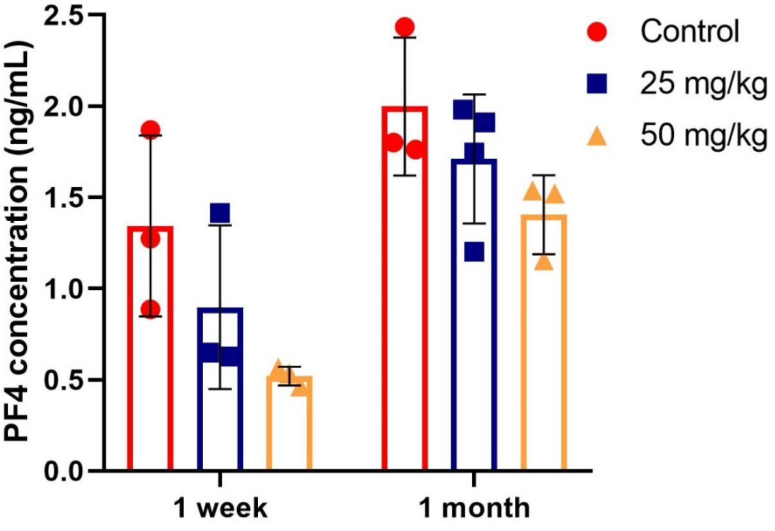

Supplementary Figure 17. PF4 concentration in blood serum determined at 1 week and 1 month post-injection. Error bars represent the standard deviation of three distinct measurements.

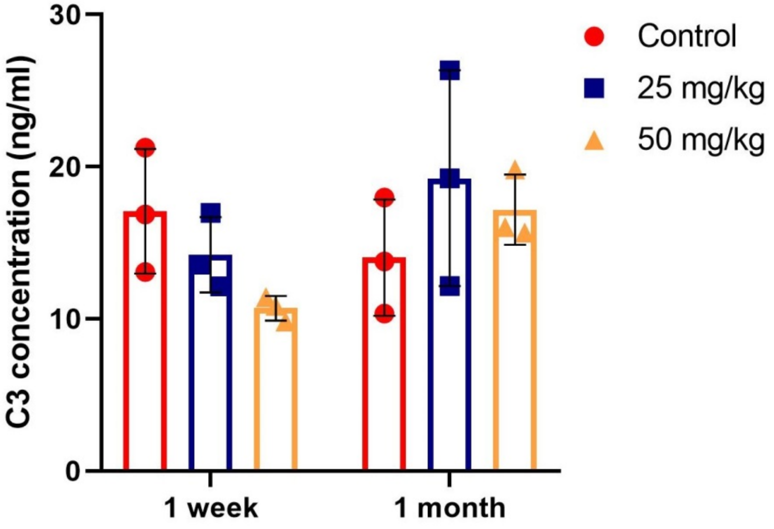

Supplementary Figure 18. C3 concentration in blood serum determined at 1 week and 1 month post-injection. Error bars represent the standard deviation of three measurements distinct.
